# Proximity Labeling and SILAC-Based Proteomic Approach Identifies Proteins at the Interface of Homotypic and Heterotypic Cancer Cell Interactions

**DOI:** 10.1016/j.mcpro.2025.100986

**Published:** 2025-05-05

**Authors:** Nazan Saner, Ceren Uzun, Büşra Aytül Kırım, Sena Nur Özkan, Daniel Jon Geiszler, Ece Öztürk, Nurcan Tunçbağ, Nurhan Özlü

**Affiliations:** 1 Department of Molecular Biology and Genetics, Koç University, İstanbul, Türkiye; 2 Department of Chemical and Biological Engineering, Koç University, İstanbul, Türkiye; 3 Koç University Research Center for Translational Medicine (KUTTAM), Koç University, İstanbul, Türkiye; 4 Department of Medical Biology, School of Medicine, Koç University, İstanbul, Türkiye

**Keywords:** cell-cell interaction, proximity labeling, SILAC, proteomics, signaling pathways

## Abstract

Cell–cell interactions are critical for the growth of organisms and maintaining homeostasis. In the tumor microenvironment, these interactions promote cancer progression. Given their importance in healthy and diseased conditions, we have developed a method to analyze the cell-to-cell interactome. Our approach uses enzyme-catalyzed proximity labeling and SILAC-based proteomics to identify the proteins involved in cancer cell interactions. By targeting HRP to the outer leaflet of the plasma membrane in bait cells, we were able to label the neighboring prey cells and distinguish between the proteomes of bait and prey cells using SILAC labeling in a coculture system. We mapped both the homotypic and heterotypic interactomes of epithelial and mesenchymal breast cancer cells. The enrichment of cell surface and extracellular proteins confirms the specificity of our methodology. We further verified selected hits from different cell–cell interactomes in cocultures using microscopy. This method revealed prominent signaling pathways orchestrating homotypic and heterotypic interactions of epithelial and mesenchymal cells. It also highlights the importance of exosomes in these interactions. Our methodology can be applied to any type of cell–cell interaction in 2D coculture or 3D tumor models.

## Highlights

• HRP-dependent biotinylation coupled with SILAC to identify cell–cell interactomes.
• Homotypic and heterotypic interactomes of breast cancer cells were profiled.
• ITGB1, ITG6, and CDCP1 from interactomes were verified in coculture.
• The method is compatible with studying cell–cell interactions in 3D culture.

## Graphical Abstract

**Figure:**
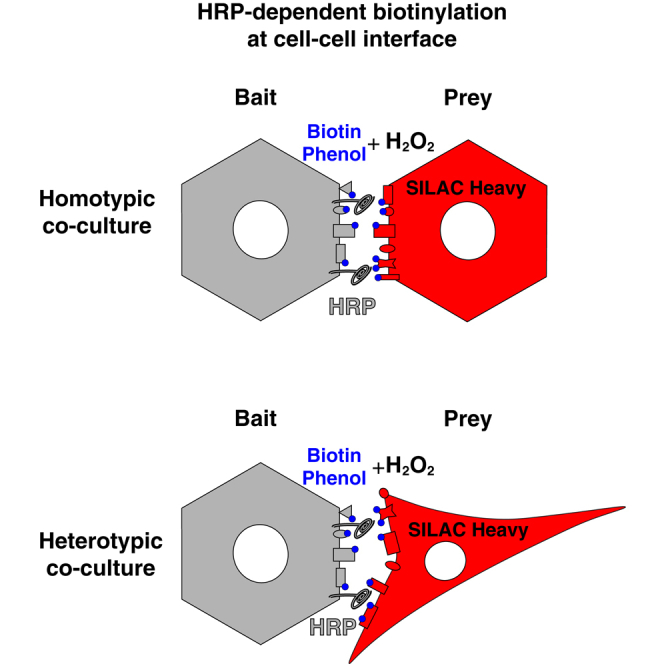

## In Brief

HRP-dependent proximity labeling was combined with SILAC-based proteomics to profile the proteome of cell–cell interface using coculture system. The homotypic and heterotypic interactomes of epithelial and mesenchymal breast cancer cells were identified. Cell surface and extracellular proteins were enriched in the interactomes. The common and unique features of different cell–cell interactions were characterized and selected hits were verified in cocultures. This methodology can be applied not only to 2D coculture systems but also to 3D tumor models.

Cellular communication plays a crucial role in maintaining homeostasis in multicellular organisms. Interaction between cells not only affects physiological processes like embryonic development and neuronal signaling but also pathological conditions like infectious diseases and cancer (1, 2, 3). Cancers show a high level of intratumor heterogeneity and possess diverse cell interactions (4, 5). A recent study using 3D organoid models derived from primary tumors identified a specialized subpopulation of cancer cells with invasive traits. The heterotypic interactions between the invasive leader cancer cells with the less invasive epithelial cancer cells drive the collective invasion (6). Another study using coculture spheroids of breast cell lines showed that the invasion-incompetent epithelial cells adjacent to the leader invasive cells get involved in collective cell migration by taking a follower role (7). Cell adhesion proteins are suggested to be involved in the heterotypic interactions of leader-follower cells. The heterophilic adhesion of N-cadherin/E-cadherin on the surfaces of leading fibroblasts and following cancer cells, respectively, was found to be crucial for the collective cell migration (8). Therefore, it is important to identify the cell surface and extracellular proteins involved in these heterotypic cell interactions to develop effective interventions to limit cancer invasion.

Although studying with monocultures of tumor-derived cell lines has given insight into the molecular mechanisms and signaling pathways involved in cancer, these cultures are unable to fully mimic the complex interactions between cancer and tumor-associated stromal cells that occur in the tumor microenvironment. *In vitro* coculture systems, on the other hand, provide a controlled environment where multiple cell lines can interact directly or indirectly in 2D or 3D arrangements with various combinations of extracellular matrix context and media (9). However, distinguishing the proteomes of cocultured cell lines has been a challenge and new methodologies were developed to overcome the limitations. For instance, trans-SILAC (trans–stable-isotope labeling of amino acids in cell culture) (10), which combines cell sorting and stable-isotope labeling by amino acids in cell culture (SILAC) (11), attempted to identify the proteins transferred between cells. Another approach is cell-selective labeling using amino acid precursors, which relies on the transgenic expression of enzymes that can synthesize essential amino acids in cocultured cell lines supplemented with isotopically labeled precursors. Labeling distinct cell populations in coculture allowed identifying the cell origin of extracellular proteins secreted from cells in the coculture (12, 13). However, none of these methods were able to identify the proximal interactions between cocultured cells.

The development of novel chemistry- and biology-based methodologies has provided opportunities to study these cell–cell interactions (14). The proteomic mapping of spatially confined regions by enzyme-catalyzed proximity labeling became a strong tool to characterize the organization and function of distinct subcellular structures and membrane-less compartments (15). Peroxidases (APEX (16), horseradish peroxidase (HRP) (17)) and biotin ligases (BioID (18), TurboID, and miniTurbo (19)) became widely used in proximity-based biotinylation. These enzymes are conjugated with a protein of interest and targeted to a specific subcellular region, where the enzyme-catalyzed reactive biotin labels only the proximal proteins. It is important to note that the proximity labeling is not limited to intracellular compartments but also applicable to extracellular regions. HRP-dependent proximity labeling was successfully applied to identify the proteome of synaptic clefts (17), which provided a strong resource to dissect the neuronal communication. Proximity labeling followed by the streptavidin pull-down of biotinylated proteins allows the enrichment of protein–protein interactions (PPI) and is orthogonal to conventional affinity purification-mass spectrometry. It is usually challenging to affinity capture proteins of insoluble compartments such as membranes. Weak and transient interactions can be also easily disrupted under harsh lysis conditions used to solubilize the membrane compartments (20). Therefore, proximity labeling offers an efficient alternative approach to affinity purification-mass spectrometry to map PPI in live cells.

In recent years, advanced proximity labeling techniques have been developed to study cell–cell interactions (14). Photocatalytic proximity labeling methods such as MicroMap (μMap) (21), LUX-MS (22), PhoTag (23), and MultiMap (24) target proteins of interest on cell surface using antibodies conjugated with a photocatalyst, which induces the protein biotinylation in the vicinity upon light stimulation. These methods offer a wide range of temporal and spatial resolution in labeling the microenvironment. In addition, the recent enzyme-based proximity labeling approaches such as LIPSTIC (labeling immune partnerships by SorTagging intercellular contacts) (25) and EXCELL (enzyme-mediated proximity cell labeling) (26) use engineered sortase A to identify receptor–ligand interactions between cells. FucoID, which relies on interaction-dependent fucosyl-biotinylation of prey cells, is not dependent on prior genetic engineering but the conjugation of α(1,3)Fucosyltransferase to LacNAc on the cell surface of bait cells by self-catalyzed chemoenzymatic labeling (27). Thus, LIPSTIC, EXCELL, and FucoID are based on contact-dependent chemical tagging to label direct cell–cell interactions, while HRP-dependent proximity labeling relies on the generation and diffusion of reactive biotin species that promiscuously tag proteins across a broader intercellular space.

In this study, we took a combinatory approach of HRP-dependent proximity biotinylation in live cells (17) and the quantitative proteomic method, SILAC (11) to biochemically analyze the interactions of cocultured cancer cells. We aimed to model the homotypic and heterotypic cell interactions in breast tumors by coculturing epithelial and mesenchymal breast cancer cell lines. We targeted HRP to the extracellular leaflet of the plasma membrane only in “bait” cells to proximity label the surface of neighboring “prey” cells in interaction. Prey cells were metabolically labeled with heavy isotope amino acids, allowing us to differentiate self-biotinylated proteins of bait cells from the prey cells. This enabled us to profile the proteins of prey cells that are interacting with the bait cells. High confident interactions between bait and prey cells were filtered by performing significance analysis of interactome (SAINT) (28, 29). The methodology's specificity was confirmed by the enrichment of cell surface and extracellular proteins in the proximity interactome. The homotypic and heterotypic interactomes of MCF7 and MDA-MB-231 cells were compared and selected hits were confirmed at cocultures by microscopy. The identified epithelial and mesenchymal proteins were functionally characterized by gene ontology (GO) enrichment analysis. Subinteraction networks were generated for enriched GO terms and compared between homotypic and heterotypic interactions. The successful proximity labeling in spheroids suggests that our methodology can be extended from 2D to 3D cocultures.

## Experimental Procedures

### Plasmids, Antibodies, and Reagents

HRP-TM (Addgene # 44441) (16) insert was cloned into pLenti CMV Hygro DEST (w117-1) (Addgene #17454) using Gateway cloning (30). The antibodies used in this study were rabbit anti-HRP (Jackson ImmunoResearch, 323-005-021) at 1:250, mouse anti-E-cadherin (Santa Cruz Biotechnology, sc-21791) at 1:50, rabbit anti-Myc (Cell Signaling Technology, 2278) at 1:1000, rabbit anti-histone H3 (Cell Signaling Technology, 4499) at 1:2000, rabbit anti-biotin (gift from Dr Timothy J Mitchison, Harvard Medical School) at 1:20,000, mouse anti-GAPDH (Cell Signaling Technology, 97166) at 1:4000, mouse anti-integrin β1 (Abcam, 30394) at 1:500, mouse anti-CDCP1 (Thermo Fisher Scientific, MA5-25357) at 1:200, rat anti-integrin α6 (Santa Cruz Biotechnology, sc-19622) at 1:50, anti-rabbit IgG HRP (Cell Signaling Technology, 7074) at 1:2000, anti-mouse IgG HRP (Cell Signaling Technology, 7076) at 1:2000, anti-rabbit IgG DyLight 488 (Invitrogen, 35552) at 1:1000, anti-rabbit IgG Alexa Fluor 555 (Cell Signaling Technology, 4413) at 1:1000, anti-mouse IgG Alexa Fluor 555 (Cell Signaling Technology, 4409) at 1:1000, anti-rat IgG Alexa Fluor 568 (Invitrogen, A-11077), Alexa Fluor 488-Streptavidin (Invitrogen, S32354) at 1:2000. Biotin phenol (BP) (Iris Biotech GmbH, LS-3500) was prepared as 1000× stock in dimethyl sulfoxide and stored at −80 °C.

### Cell Culture, SILAC Labeling, Stable Cell Line Generation, Coculture, and Spheroid Preparation

MCF7 and MDA-MB-231 cells were maintained in Dulbecco's Modified Eagle's Medium (DMEM) high glucose supplemented with 10% fetal bovine serum (FBS), 1% penicillin–streptomycin, and 1% MEM nonessential amino acids. For coculture experiments, SILAC labeling was performed in MCF7 and MDA-MB-231 cells. Cells were grown in SILAC-DMEM (Thermo Fisher Scientific, 88364) supplemented with 10% dialyzed FBS (Lifesciences, 26400044), 1% penicillin–streptomycin, 1% MEM nonessential amino acids, L-Lysine:2HCL (^13^C_6_, ^15^N_2_), and L-Arginine:HCL (^13^C_6_, ^15^N_4_) (Cambridge Isotope Laboratories, CNLM-291 and CNLM-539) at concentrations of 0.8 mM and 0.4 mM, respectively. All cells were grown at 37 °C in a humidified atmosphere containing 5% CO_2_. The incorporation of heavy labeled amino acids was assured by at least 10 passages.

For the generation of HRP-TM–expressing stable cell lines, MCF7 and MDA-MB-231 cells were transduced with lentiviral particles. Briefly, HEK293T cells were transfected with psPAX2 (Addgene #12260), pCMV-VSV-G (Addgene #8454), (31) and HRP-TM lentiviral vector using Lipofectamine 2000 transfection reagent (Invitrogen 11668027) according to the manufacturer's instructions. The viral supernatants were collected at 48- and 72-h after transfection and filtered through a 0.22 μm filter. The cells were infected with the pooled viral supernatants in medium supplemented with 2 μg/μl protamine sulfate (Sigma-Aldrich P4505) as coadjuvant. The virus-infected MCF7 and MDA-MB-231 cells were selected with 150 μg/ml and 400 μg/ml hygromycin (Sigma-Aldrich, H3254) treatment, respectively for 1 week. MDA-MB-231-luc2-GFP cells were a kind gift from Dr Özgür Şahin, Medical University of South Carolina.

For the spheroid preparation, 15 mg/ml alginate (NovaMatrix) was dissolved in serum-free DMEM/F12 and sterile filtered using a 0.2 μm pore size syringe filter. Following this, 100 μl of cell suspension (50 million cells/ml) comprising HRP-TM–expressing and WT MCF7 cells in a 1:1 ratio was combined with 200 μl of the alginate solution. The resultant mixture was used to generate alginate beads using a 5 ml 22G syringe, deposited into a sterile 100 mM CaCl_2_-containing beaker placed on a magnetic stirrer. After 5 min of gelation, the CaCl_2_ solution was decanted through a cell strainer to recover the beads. These beads were then seeded into the wells of a 48-well plate and incubated for 18 h to facilitate the formation of cellular spheroids.

### HRP-Based Proximity Labeling and Affinity Capture of Labeled Proteins

For monocultures, two confluent 15 cm dishes were grown before proximity labeling with BP. For homotypic and heterotypic cocultures, two confluent 10 cm dishes of HRP-TM (+) cells and four confluent 10 cm dishes of heavy labeled cells were mixed. The confluent 10 cm plates of MCF7 and MDA-MB-231 correspond to approximately 3 × 10^6^ and 1.5 x 10^6^ cells, respectively. The mixed cells were centrifuged at 1000 rpm for 5 min. After the medium was aspirated, the pellet was resuspended in regular medium. Cells were equally divided and seeded onto two 15 cm dishes and incubated for 18 h to establish cell–cell interactions. Peroxidase-based protocol was followed for the proximity labeling (32). Briefly, one dish was incubated with regular medium supplemented with 500 μM BP and 1 mM H_2_O_2_ at room temperature (RT) for 1 min. The control plate was only treated with dimethyl sulfoxide and 1 mM H_2_O_2_. The medium was quickly aspirated, and cells were washed twice with quencher solution (10 mM sodium ascorbate, 5 mM trolox, 10 mM sodium azide solution in PBS), then twice with PBS and one more time with quencher solution. After scraping cells with quencher solution, cells were pelleted by centrifugation for 10 min at 1500 rpm at 4 °C. The cell pellets were stored at −80 °C after flash freezing with nitrogen.

The cell pellet of one 15 cm plate was resuspended in 800 μl of radioimmunoprecipitation assay (RIPA) lysis buffer (50 mM Tris–HCl pH 7.5, 150 mM NaCl, 0.1% SDS, 0.5% sodium deoxycholate, and 1% Triton X-100) supplemented with Pierce EDTA-free protease inhibitor (Thermo Fisher Scientific, A32955), 1 mM PMSF (Cayman, 14,333), and quenchers (10 mM sodium azide, 10 mM sodium ascorbate, and 5 mM Trolox). After resuspension, the samples were kept on ice for 2 min. The lysates were centrifuged at 15,000 g for 10 min at 4 °C and the cleared supernatants were transferred into fresh tubes. The protein concentration was measured using the Pierce 660-nm assay (Thermo Fisher Scientific, 22660). For each sample, 175 μl aliquots of slurry streptavidin beads (Pierce Streptavidin UltraLink Resin, 53117) were washed twice with 1 ml of RIPA lysis buffer. Each cell lysate was incubated with streptavidin beads at 4 °C overnight on a rotator. Then, each bead sample was washed twice with RIPA lysis buffer, once with 1 M KCI, once with 0.1 M Na_2_CO_3_, once with 2 M urea in 10 mM Tris–HCl pH 8.0, and twice with RIPA lysis buffer to reduce nonspecific binding. All the washes were performed on ice. Ten percentage of beads were spared for Western blot analysis and biotinylated proteins were eluted from the beads by incubating with 2× Laemmli buffer supplemented with 10 μM D-biotin and 100 mM DTT at 95 °C for 10 min.

### On-Bead Trypsin Digestion of Labeled Proteins and Desaltation of Peptides

Each bead sample was washed twice with 100 mM Tris–HCl pH 8.5 and twice with 8 M urea in 100 mM Tris–HCl pH 8.5 (urea solution). After the disulfide bonds of proteins on beads were reduced with 100 mM DTT in urea solution for 30 min of shaking at 1000 rpm, at 56 °C, beads were washed with urea solution. Cysteine alkylation was achieved with 50 mM chloroacetamide in urea solution for 20 min of shaking at 1000 rpm, at 25 °C. Beads were then washed twice with urea solution and twice with 50 mM ammonium bicarbonate. Pierce MS grade trypsin (PI-90057, Thermo Fisher Scientific) was added on beads at 1:100 protease:protein ratio. After incubation on ice for 15 min, 500 μl of ammonium bicarbonate was added on beads. The protein digest was carried out overnight by shaking beads at 1000 rpm, at 37 °C. After centrifugation, the peptides (supernatant) were transferred to a fresh tube. Beads were further incubated with 300 μl of ammonium bicarbonate for 10 min and the supernatant was pooled with the first one. The digestion was quenched with 10% formic acid (FA) to a final concentration of 1% FA. The peptides were dried by vacuum centrifugation at RT and stored at −20 °C.

The dried peptides were reconstituted in 1 ml of 0.1% FA and sonicated for 10 min. Sep-Pak C18 1 cc Vac cartridges (Waters, WAT023590) were conditioned with 1 ml acetonitrile (ACN) and washed twice with 0.1% FA. After the Sep-Pak C18 cartridges were loaded with samples, they were washed twice with 0.1% FA. The peptides were eluted with 1 ml of 0.1% FA in 80% ACN and dried by vacuum centrifugation at RT. The samples were stored at −20 °C before MS analysis.

### LC-MS/MS Analysis

The samples were reconstituted in LC/MS-MS loading buffer (5% ACN and 5% FA) and analyzed by reversed phase nanoflow liquid chromatography system (Ultimate 3000 RSLCnano, Thermo Fisher Scientific) coupled to Q Exactive or Q Exactive HF Orbitrap mass spectrometer (Thermo Fisher Scientific). Peptides were loaded onto an in-house packed 75 μm i.d. × 45 cm C18 reversed-phase column (Reprosil-Pure C18, 1.9 μm, 200 Å, Dr Maisch) and run with a flow rate of 300 nl/min with 100 min linear gradients, increasing from 5% to 40% ACN in 0.1% FA within a 120 min total run time. Peptides in the mass range of 400 − 1500 m/z were allowed to be detected in Orbitrap in data-dependent acquisition mode. For Orbitrap MS1 scanning, the resolution was set to 70,000, the automatic gain control target to 3e6, and the maximum injection time to 60 ms. The top 15 most intense peptides per cycle were selected and fragmented in the higher-energy collisional dissociation cell with a normalized collision energy of 26. For Orbitrap MS2 detection, the resolution was set to 17,500, the automatic gain control target to 1e5, and the maximum injection time to 60 ms. The isolation window for MS/MS was set to 2.0 m/z. As a data-dependent setting, dynamic exclusion was set to 50 s, and charge exclusion was set to unassigned, 1, >6.

Multiple technical replicates for each biological replicate were processed as Multidimensional Protein Identification Technology (MudPIT) samples using Proteome Discoverer Daemon utility. MudPIT files were analyzed by Proteome Discoverer (PD) (v2.3, Thermo Fisher Scientific) using Sequest HT search engine for peptide identification and quantification. The search was performed against the human proteome sequences retrieved from UniProt (21,046 entries, March 2016), including HRP-TM. Carbamidomethylation of cysteine was set as fixed modification. Biotinylation of tyrosine by BP (C_18_H_23_N_3_O_3_S), acetylation of protein N-termini, and oxidation of methionine were set as variable modifications. L-Lysine:2HCL (^13^C_6_, ^15^N_2_) and L-Arginine:HCL (^13^C_6_, ^15^N_4_) were introduced as variable modifications only for coculture samples. A maximum of two missed cleavages was allowed for the tryptic peptides. A mass tolerance of ±10 ppm for precursor masses and ±0.02 Da for fragments ions were selected. Both peptide and protein false discovery rates were set to 1%. Peptides were filtered by medium or high identification confidence and peptides with a sequence length between 7 and 25 amino acids.

### Data Analysis

The pair-wise Pearson correlation coefficient of three biological replicates for each condition was calculated using the peptide-spectrum match (PSM) counts. For cocultures, only heavy labeled peptides of prey cells were selected, and their PSM counts were summed up for each protein. SAINTexpress analysis (28, 29) of three biological replicates was performed to eliminate the false positive interactions. Interactions with SAINT score>0.7 and Bayesian false discovery rate < 0.05 were accepted as high confidence. Following that, the identified interactome for each condition was categorized by five GO cellular compartment terms (33); GO:0030054 (cell junction), GO:0005886 (plasma membrane), GO:0005615 (extracellular space), GO:0005737 (cytoplasm), and GO:0005634 (nucleus) using the QuickGo web tool (34). For multiple annotations, the priority was given in the former order. Proteins that were annotated to cell junction, plasma membrane, and extracellular space were enriched for GO biological processes, molecular functions, and cellular component (CC) using the Cytoscape plug-in ClueGo (v2.5.10) (35). Bonferroni step-down method was used to correct the *p*-values for multiple testing. Protein networks were generated using the previously identified high-confidence protein–protein interactions and the STRING database (v12) (36). Networks were visualized using Cytoscape (v3.8.2) (37). The heat maps were generated using GraphPad Prism 10. Area-proportional Venn diagrams were created with DeepVenn (38).

### Experimental Design and Statistical Rationale

We designed the experiment to identify the whole cell surface proteome of epithelial and mesenchymal cancer cells and the proteins at the interface of homotypic and heterotypic interactions of these cancer cells. For this, three biological replicates of monoculture, homotypic, and heterotypic cocultures of MCF7 and MDA-MB-231 cells were prepared. Each biological replicate contained a BP and H_2_O_2_-treated culture and only H_2_O_2_-treated culture as control.

### Immunofluorescence Staining and Microscopy

Cells were grown on glass coverslips and proximity labeled with BP as described above. Cells were fixed with either 10% trichloroacetic acid for 10 min at 4 °C or 4% paraformaldehyde (PFA) in PBS for 15 min at RT and washed three times with PBS for 5 min. After blocking with 2% bovine serum albumin (BSA) in PBS, cells were incubated with primary antibody in blocking solution either overnight at 4 °C or 3 h at RT. Following three times PBS washes, cells were incubated with secondary antibody and Alexa Fluor 488-Streptavidin (Invitrogen, S-32354) in blocking solution for 1 h at RT. After three times PBS washes, DNA was stained with either 4′,6-diamidino-2- phenylindole (1 μg/ml) or Hoescht (1 μg/ml) in blocking solution for 10 min at RT and cells were washed three times with PBS. Samples were embedded in Mowiol mounting medium (Sigma-Aldrich, 81381). The images were acquired using 63× Plan Apo 1.4 NA oil-immersion objective of Leica DMi8/SP8 (LAS X Software) laser scanning confocal microscope or 63× Plan Apo 1.4 NA oil-immersion objective of Leica DMi8 wide-field microscope.

Spheroids were grown in 48-well plates and proximity labeled with BP as described above. They were fixed with 4% PFA in wash buffer (100 mM NaCl, 5 mM CaCl_2_) for 45 min at 4 °C and washed twice with wash buffer for 15 min. After the alginate beads were blocked with 2% BSA in wash buffer for 1 h at RT, they were incubated with primary antibody in blocking solution overnight at 4 °C. Following two washes with wash buffer for 30 min each, the beads were incubated with secondary antibody and Alexa Fluor 488-Streptavidin (Invitrogen, S-32354) in blocking solution for 2 h at RT. After two additional washes, DNA was stained with 4′,6-diamidino-2- phenylindole or Hoescht in blocking solution for 15 min at RT. The beads were washed twice with wash buffer for 10 min and placed onto a glass coverslip bottom dish (ibidi μ-Dish 35 mm low) for microscopy imaging. The images were acquired using the 20×HC PL FLUOTAR 0.4 NA objective of Leica DMi8 wide-field microscope or 20×HC PL FLUOTAR 0.55 NA objective of Leica DMi8/SP8 (LAS X Software) laser scanning confocal microscope. The images were processed using Fiji software (39) and single z-section images were shown in figures.

### FACS Analysis

MCF7 HRP-TM and MDA-MB-231 GFP cells were cocultured in 1:1 ratio in 15 cm dishes and proximity labeled with BP as described above. Negative control coculture was only treated with H_2_O_2_ and BP was omitted. Coculture of MCF7 HRP-TM and MDA-MB-231 cells was also established to set the GFP gate for fluorescence-activated cell sorting (FACS). After biotinylation, cells were scraped with 5 mM EDTA, 25 mM Hepes, 1% FBS in PBS (PBS buffer) and pelleted by centrifugation for 5 min at 300*g* at 4 °C. The cell pellets were first resuspended in 500 μl of cold PBS buffer and mixed with 500 μl of cold 2% PFA in PBS buffer. The cell fixation took place for 15 min at 4 °C, followed by three times wash with cold PBS buffer. Cells were filtered through 70 μm nylon mesh before FACS. Proximity labeled and control cocultures (MCF7 HRP-TM and MDA-MB-231 GFP cells) were sorted based on their GFP signal by SONY SH800 Cell Sorter. One lakh events were acquired for each condition.

### Western Blotting

Cell pellets were resuspended in RIPA lysis buffer supplemented with protease inhibitors and quenchers. Proteins were separated by 10% SDS–PAGE and transferred onto nitrocellulose membranes. After blocking with 4% milk in TBS-0.1% Tween20 (TBS-T) for 1 h at RT, the blots were incubated with primary antibodies in 2% BSA in TBS-T overnight at 4 °C, followed by three times TBS-T rinse for 5 min. The blots were incubated with HRP-conjugated secondary antibodies at RT for 1 h and rinsed with TBS-T three times for 5 min. The blots were then visualized using the Pierce ECL Western Blotting Substrate system (Thermo Fisher Scientific, 32106) and X-ray films or ChemiDoc imaging system (Bio-Rad). Signal quantification was performed using Fiji software (39).

## Results

### Cell Surface–Localized HRP is Able to Biotinylate the Proximal Proteins of Neighboring Cells

**Fig. 1:**
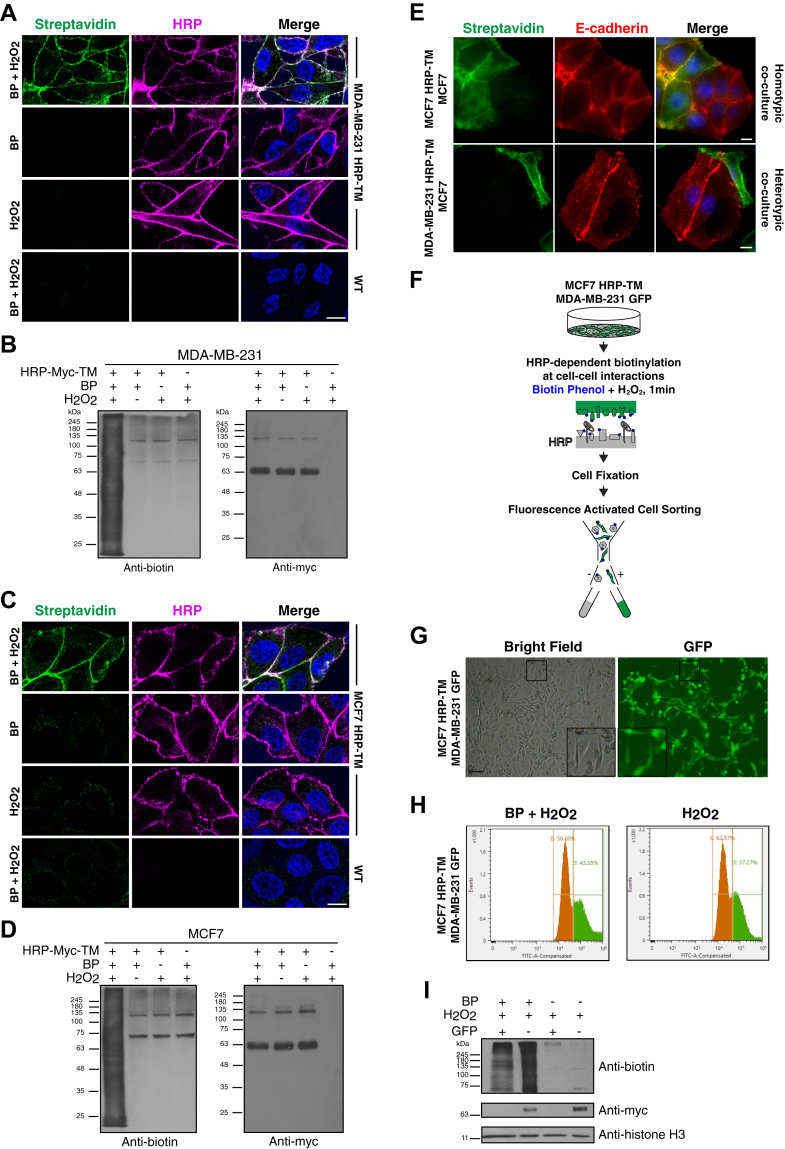
**HRP that is expressed on the surface of breast cancer cells can biotinylate the proximal proteins of neighboring cells.***A* and *C*, the representative immunofluorescence images of HRP-TM expressing stable MDA-MB-231 cells (MDA-MB-231 HRP-TM) and HRP-TM expressing stable MCF7 cells (MCF7 HRP-TM) after biotinylation, respectively. HRP-TM–expressing cells treated with either BP or H_2_O_2_ and WT cells treated with BP and H_2_O_2_ were used as controls. Cells were fixed and stained with fluorescent streptavidin for biotinylation and with anti-HRP antibody. DNA was stained with Hoescht. Scale bar represents 10 μm. *B* and *D*, the specific biotinylation of MDA-MB-231 HRP-TM and MCF7 HRP-TM cells was tested under different conditions with Western blot analysis, respectively. Cell lysates were immunoblotted with antibodies against biotin and myc (for HRP-Myc-TM). *E*, representative immunofluorescence images of homotypic and heterotypic interactions that were established by coculturing MCF7 HRP-TM with MCF7 and MDA-MB-231 HRP-TM with MCF7 cells, respectively. After biotinylation, cells were fixed and stained with fluorescent streptavidin for biotinylation and with anti-E-cadherin antibody to visualize MCF7 cells. DNA was stained with DAPI. Scale bar represents 10 μm. *F*, experimental workflow to validate that HRP-TM expressing cells can biotinylate the neighboring cells. MCF7 HRP-TM and GFP-expressing stable MDA-MB-231 (MDA-MB-231 GFP) cells were cocultured. Upon biotinylation and cell fixation, MCF7 HRP-TM and MDA-MB-231 GFP cells were sorted. *G*, representative bright and fluorescence images of MCF7 HRP-TM/MDA-MB-231 GFP coculture. Scale bar represents 50 μm. *H*, sorting of BP-treated or control MCF7 HRP-TM/MDA-MB-231 GFP cocultures after cell fixation. *I*, lysates of sorted cells were immunoblotted with antibodies for biotin, myc (HRP-Myc-TM), and histone H3 (loading control).

To study homotypic and heterotypic cancer cell interactions, the epithelial and mesenchymal breast cancer cell lines MCF7 and MDA-MB-231, respectively, were chosen to mimic the intratumor heterogeneity of breast cancer. To identify the proteome involved in cancer cell interactions, the HRP-conjugated transmembrane domain (21 amino acid) of platelet-derived growth factor receptor (HRP-TM) (16), which is expressed all over the external side of the plasma membrane, was employed for proximity labeling. The biotinylation specificity of HRP-TM on the surface of MDA-MB-231 and MCF7 cells were confirmed with immunofluorescence staining. Cell surface–specific biotinylation was not observed in MDA-MB-231 HRP-TM and MCF7 HRP-TM cells treated with only BP or H_2_O_2_, nor in WT cells treated with BP and H_2_O_2_ (Fig. 1, *A* and *C*). Biotinylation efficiency and specificity were also verified with Western blotting. Proximity-based biotinylation was dependent on the expression of enzyme and the supplements of BP and H_2_O_2_. Only endogenously biotinylated proteins (40) were observed in the absence of enzyme and supplements (Fig. 1, *B* and *D*).

As this study aims to identify the proteome of cell–cell interactions, cocultures of HRP-TM–expressing “bait” cells and regular “prey” cells were established to model homotypic and heterotypic interactions (Fig. 1*E*). While the whole cell surfaces of bait cells expressing HRP-TM were biotinylated, the prey cells did not show whole surface biotinylation. To determine whether the bait cells could biotinylate the proximal prey cells *via* the HRP-TM on their surface, we coupled the proximity labeling of cocultures with FACS analysis, which allows distinguishing the bait and prey cells. For this, the coculture of HRP-TM–expressing bait cells (MCF7 HRP-TM) and GFP-expressing prey cells (MDA-MB-231 GFP) were proceeded with proximity biotinylation (Fig. 1, *F* and *G*). Following fixation, bait and prey cells were sorted based on the GFP signal intensity by FACS analysis (Fig. 1, *F* and *H*). Our Western blotting analysis revealed that GFP-expressing prey cells were successfully biotinylated by the HRP-TM–expressing bait cells only when cells were supplemented with BP (Fig. 1*I*).

### HRP-Dependent Proximity Labeling and SILAC were Combined to Map the Proteome at the Interface of Homotypic and Heterotypic Cancer Cell Interactions

**Fig. 2:**
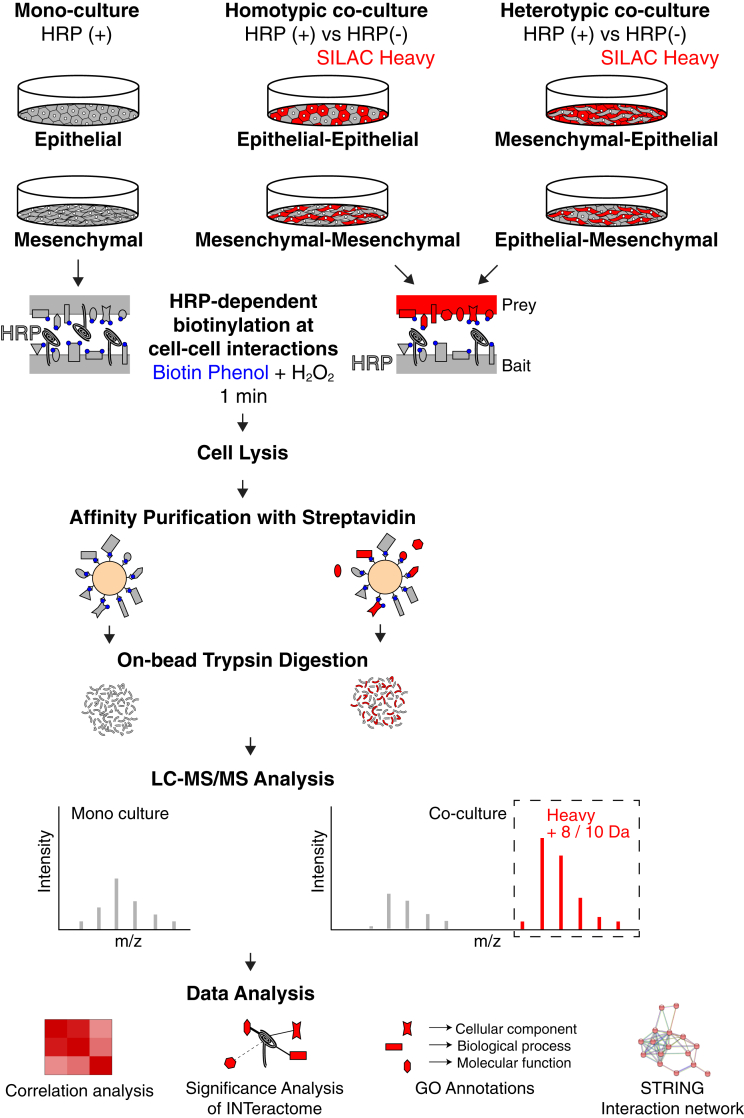
**Schematic representation of the integrative proteomic approach that combines SILAC and HRP-dependent proximity labeling to identify proteins involved in distinct cell–cell interactions.** Homotypic (same cell) and heterotypic (different cells) cocultures are established. HRP targeted to the cell surface in the bait subpopulation biotinylates the proximal proteins in prey subpopulation. Monocultures are set to identify the whole surface proteome of epithelial and mesenchymal cell lines. Upon proximity labeling, cells are lysed and biotinylated proteins are affinity purified. Prior SILAC labeling allows the specific identification of prey proteins in cocultures by mass spectrometry. Bioinformatic analysis enables us to distinguish high confident interactions between bait and prey cells and characterize the prey proteins in terms of gene ontologies.

**Fig. 3:**
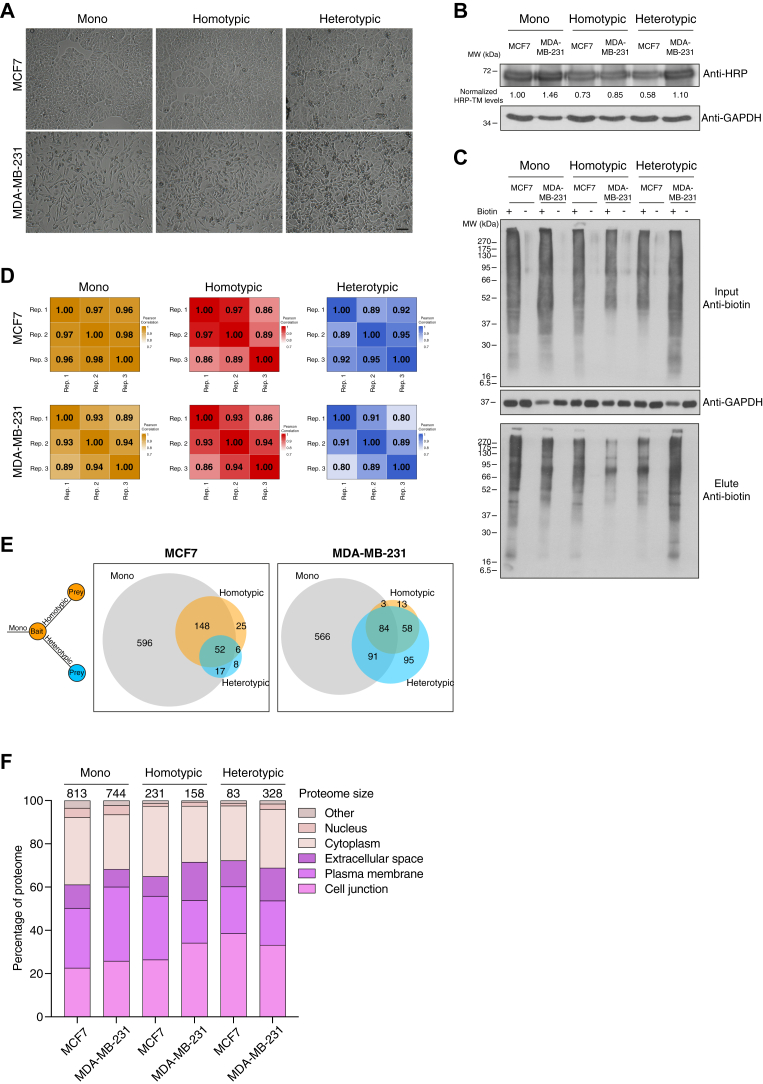
**Cell surface and extracellular proteins were enriched at the cell–cell interactome***A*, the representative bright field images of monoculture and cocultures that were established in 18 h. Scale bar represents 50 μm. *B*, HRP-TM levels in monoculture and cocultures (heterotypic MCF7 coculture (MDA-MB-231 HRP and MCF7) and heterotypic MDA-MB-231 coculture (MCF7 HRP and MDA-MB-231)). Whole cell lysates were immunoblotted with antibodies against HRP and GAPDH (loading control). *C*, affinity capture of biotinylated proteins using lysates of monocultures, homotypic, and heterotypic cocultures of epithelial and mesenchymal cell lines treated with BP and H_2_O_2_ or only with H_2_O_2_ for 1 min. Input and streptavidin pull-down samples were immunoblotted with antibodies against biotin and GAPDH (loading control). *D*, the pair-wise Pearson correlation of biological replicates for monocultures, homotypic, and heterotypic cocultures of MCF7 and MDA-MB-231. *E*, Venn diagram shows the number of high confidence interactions identified for monocultures, homotypic, and heterotypic cocultures of MCF7 and MDA-MB-231, n = 3 biological replicates. The comparison of proximity interactomes at each condition reveals the level of overlaps and distinctions. *F*, the categorization of identified proteins into five GO CC terms using QuickGo web tool. The priority was given in the following order for multiple annotations: Cell junction > plasma membrane > extracellular space > cytoplasm > nucleus.

Our data demonstrated the efficient biotinylation of interacting prey cells by HRP-TM–expressing bait cells. We then combined this approach with SILAC metabolic labeling to distinguish the proteomes of bait and prey cells. In this experimental setup (Fig. 2), the prey cells, which did not possess HRP-TM, were metabolically labeled with heavy amino acids to differentiate their proteomes from the bait cells. Confluent homotypic and heterotypic cocultures of epithelial and mesenchymal cells were established at around 18 h in a regular culture medium (Experimental Procedures) to minimize the dilution of heavy labeling in prey cells. To enhance the interaction of seed bait cells and the surrounding prey cells in cocultures, bait and prey cells were mixed in an approximately 1:2 ratio based on the surface area they occupy. The overview bright field images of cocultures reveal the establishment of cell–cell contacts in 18 h (Fig. 3*A*). As an epithelial cell line, MCF7 cells form tight junctions and grow in high numbers in a limited space. In contrast, MDA-MB-231 cells tend to spread out more; upon reaching confluency, they stop growing and frequently round up (Fig. 3*A*). When HRP-TM levels were monitored in monocultures and cocultures by Western blotting (Fig. 3*B*), both MCF7 HRP and MDA-MB-231 HRP cells in monocultures exhibit similar levels of HRP expression. However, since the number of MDA-MB-231 cells in a given area is approximately half that of MCF7 cells (Experimental Procedures), we observed a difference in HRP-TM levels in heterotypic cocultures. This is not the case in homotypic cocultures, in which bait and prey cells are the same type (Fig. 3*B*).

After establishing the cell–cell interactions, BP and H_2_O_2_ were simultaneously added to achieve HRP-dependent biotinylation for 1 min. Cells were not pre-incubated with BP to prevent its intracellular uptake. HRP-dependent biotinylation in monocultures of epithelial and mesenchymal cell lines was performed in parallel to identify the whole cell surface proteome of individual cell lines and to discriminate the cell surface proteins that were not involved in cell–cell interactions. Following the proximity labeling, cells were lysed and biotinylated proteins were affinity purified using streptavidin beads. The efficiency of both biotinylation and the pull-down assay were confirmed by Western blotting. Monocultures or cocultures that were treated with only H_2_O_2_ and not with BP were processed as controls (Fig. 3*C*).

Three biological replicates were performed to map the whole surface proteome of cancer cells (monocultures) and the proteome of homotypic and heterotypic cancer cell interactions (cocultures). The eluates were run on an Orbitrap mass spectrometry and analyzed with Proteome Discoverer (PD) (supplemental Table S1). LC-MS/MS analysis of peptides identified all the purified proteins that were biotinylated both in bait and prey cells. Setting heavy labeling as a variable modification during analysis allowed us to distinguish the prey proteins that were proximal to HRP-TM on the surface of bait cells in cocultures. At this step, light peptides were discarded to isolate the signal from prey proteins for further analysis (Fig. 2). The PSM counts from three biological replicates were subjected to correlation analysis for each condition. The pair-wise Pearson correlation coefficients range from 0.8 to 0.97 (Fig. 3*D*). The overall distribution of PSM counts per protein for each experiment is shown in supplemental Fig. S1. Based on the PSM counts of samples and controls, SAINTexpress analysis (28, 29) assigned a confidence score for each PPI, and interactions with SAINT score>0.7 and Bayesian false discovery rate< 0.05 were selected for further analysis (supplemental Table S2 and supplemental Fig. S2). A higher number of proteins were identified in monocultures than cocultures. MS analysis detected 813 and 744 proteins on the surface of MCF7 and MDA-MB-231 cells in monocultures, respectively. 87% and 83% of proteins involved in homotypic and heterotypic interactions of MCF7 overlapped with the cell surface proteome of MCF7 in monocultures, respectively. However, these ratios dropped to 55% and 53% in the case of MDA-MB-231 cells (Fig. 3*E*). This suggests that more unique proteins were involved in cell–cell interactions of MDA-MB-231 cells that were masked in the whole cell surface proteome of monocultures. To address the enrichment of cell surface–related proteins for each condition, the identified proteins were mainly categorized into five broad GO CCs (33): cell junction, plasma membrane, extracellular space, cytoplasm, and nucleus. For multiple annotations, the priority was given in the former order. The collective ratio of proteins from the cell surface and extracellular space at homotypic and heterotypic interactions range from 65% to 72% (Fig. 3*F*). The percentages of nuclear proteins are very low and negligible compared to the other CCs. Cytoplasmic proteins can be the interaction partners of plasma membrane–associated proteins in large complexes, which might be collectively pulled down. Overall, our methodology is able to monitor the cell–cell interfaces.

### The Proteomic Comparison of Homotypic and Heterotypic Interactions of Epithelial and Mesenchymal Cancer Cells Reveals Common and Distinct Features

**Fig. 4:**
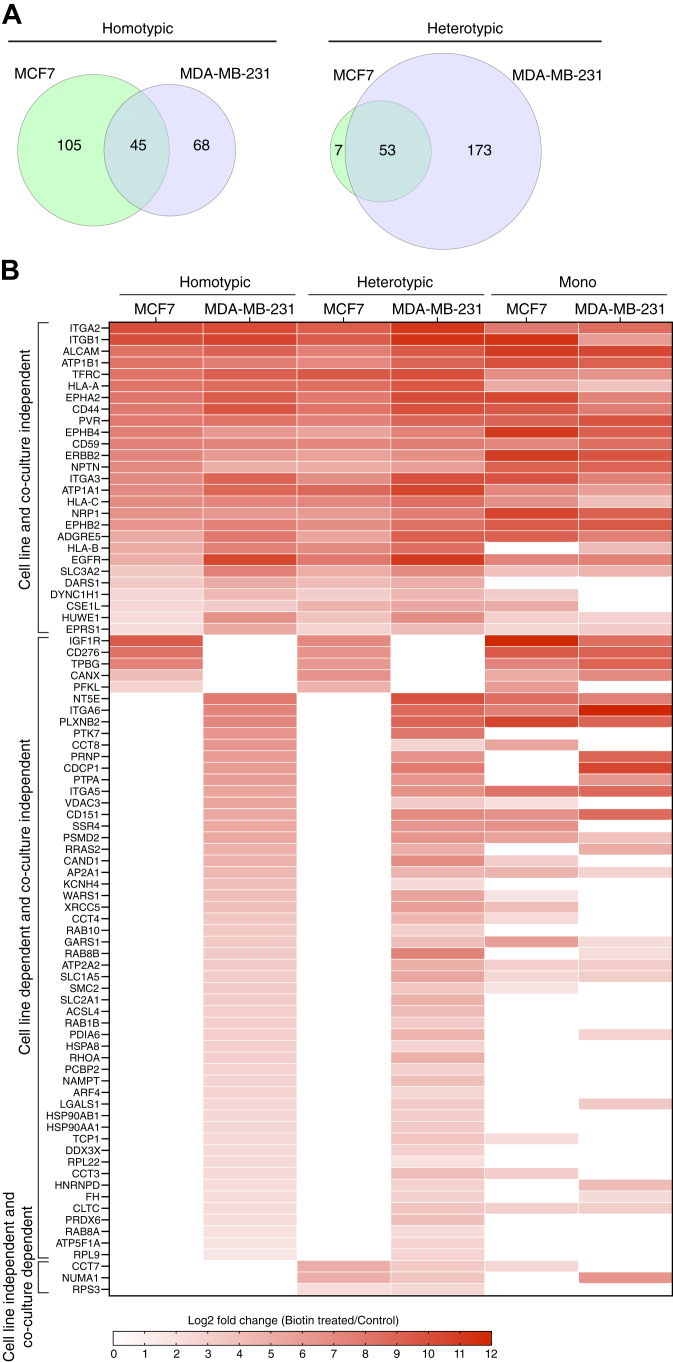
**The common and distinct features of epithelial and mesenchymal proteins at homotypic and heterotypic interactions.***A*, Venn diagram shows the number of common, MCF7 and MDA-MB-231 selective proteins at homotypic and heterotypic interactions. *B*, representative clusters that were generated based on the identification pattern of proteins at homotypic and heterotypic interactions of MCF7 and MDA-MB-231. The rest of the clusters are presented in supplemental Fig. S3. Heatmap reveals the log_2_ fold changes of identified proteins in comparison to controls.

Because we are interested in the surface-exposed proteins of the prey cells, we filtered the nucleus- and cytoplasm-annotated proteins out for further analysis. When we compared the proteome at the interface of homotypic interactions of epithelial and mesenchymal cancer cells, we observed 45 proteins in common between MCF7 and MDA-MB-231 cells, which corresponds to 30 to 40% overlap along with 105 and 68 proteins selective for MCF7 and MDA-MB-231 cells, respectively. However, the majority of identified proteins in heterotypic interactions of MCF7 cells (88%) overlapped with the proteome of MDA-MB-231. In contrast, 76% of identified proteins in heterotypic interactions of MDA-MB-231 cells were found to be selective for MDA-MB-231 (Fig. 4*A*). This data is in line with the Western blotting analyses (Fig. 3, *B* and *C*), which reveal higher HRP-TM levels and biotinylation in heterotypic interactions of MDA-MB-231 compared to MCF7 cells, due to discrepancy in the number of cells cocultured as discussed above.

To examine the common and unique features of the cell–cell interactome, we categorized the proteins based on their identification patterns under different coculture conditions in epithelial and mesenchymal cell lines. The heat map revealed not only the distinct clusters of proteins identified at cell–cell interactions but also the fold changes of proteins in comparison to the control cells (Fig. 4*B*). One group represents the common cell–cell interactome that were identified in each cell line and under each coculture condition, which may be canonical players in various cell–cell interactions across different cells and physiological contexts. For instance, HLA class 1a molecules (HLA-A, -B, and -C), which are antigen-presenting transmembrane proteins on the surface of most nucleated cells (41), are present in this group. Similarly, some members of integrins (ITGA2, ITGB1, ITGA3) and cell surface receptors (CD44 and EGFR) are core interactome proteins present in all conditions. Another group involves the proteins that were identified only in a specific cell line independent of coculture conditions. Integrin α6 (ITGA6), a transmembrane adhesion receptor that mediates cell-extracellular matrix and cell-cell adhesion (42), and CUB domain-containing protein (CDCP1), a cell surface glycoprotein (43), were only identified at homotypic and heterotypic interactions of MDA-MB-231 cells. In line with this, both were shown to be highly expressed in MDA-MB-231 cells in comparison to MCF7 cells and their high expression was linked to the metastatic potential of cell lines (44, 45, 46). Only three proteins (CCT7, NUMA1, and RPS3) are cell line–independent, but coculture-dependent, and were only identified under heterotypic cocultures (Fig. 4*B*). Based on their identification patterns, the rest of the proteins were grouped in eight distinct clusters (supplemental Fig. S3).

### Verification of Selected Proteins from Cell–Cell Interactomes by Imaging Cocultures

**Fig. 5:**
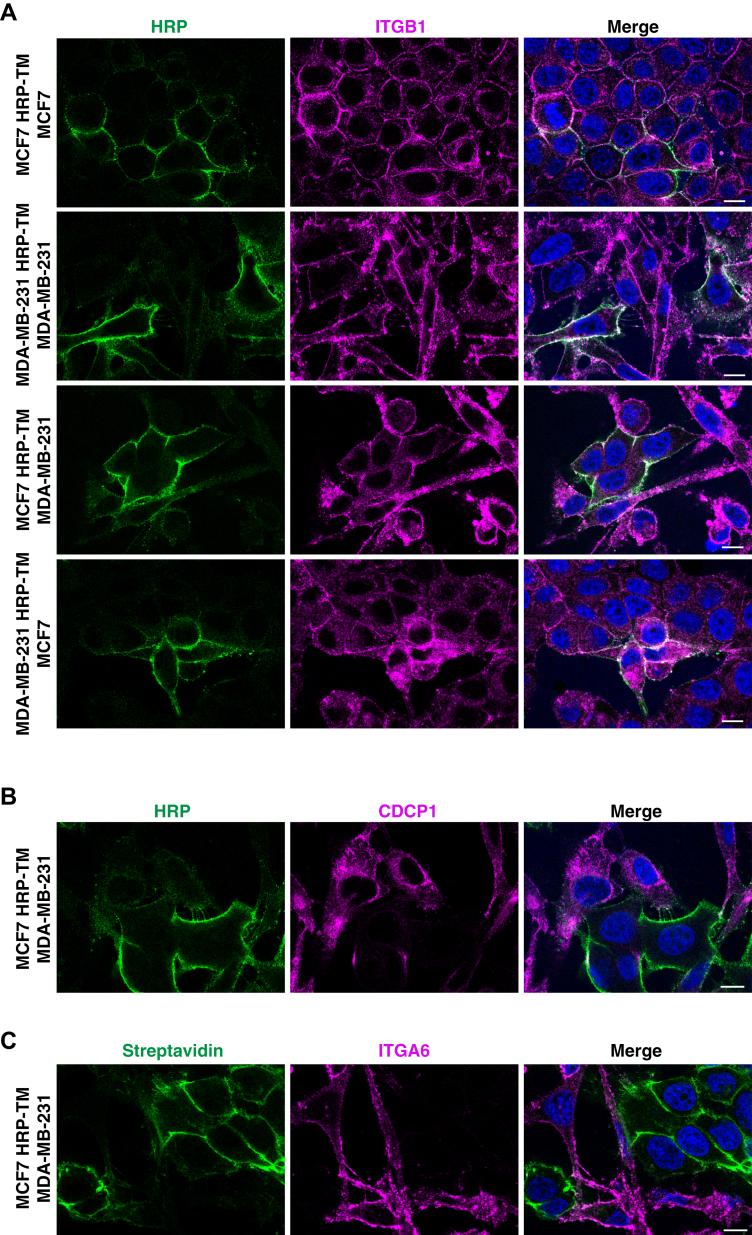
**The overview images of cocultures and validation of cell–cell interactomes.***A*, the representative immunofluorescence images of homotypic and heterotypic cocultures of MCF7 and MDA-MB-231 cells. Cells were fixed and stained with anti-HRP antibody to visualize bait cells and with anti-ITGB1 antibody. *B*, the representative immunofluorescence image of MCF7 HRP-TM and MDA-MB-231 coculture. Cells were fixed and stained with anti-HRP antibody to visualize MCF7 HRP-TM cells and with anti-CDCP1 antibody. *C*, the representative immunofluorescence image of MCF7 HRP-TM and MDA-MB-231 coculture that was proximity labeled for 1 min. After biotinylation, cells were fixed and stained with fluorescent streptavidin for biotinylation and with anti-ITGA6 antibody. DNA was stained with Hoescht and scale bar is 10 μm in all images.

We then tested representative proteins from different cell–cell interactome patterns by imaging them in coculture conditions that were established in 18 h. Although integrin β1 (ITGB1) seems to be expressed more on the cell surface of MDA-MB-231 cells, it colocalized with HRP in both homotypic and heterotypic interactions of MCF7 and MDA-MB-231 cells as expected (Fig. 5*A*). Next, we set up a time series for the coculture of MCF7 HRP-TM and MDA-MB-231 cells. The establishment of cell–cell contacts was seen from 12 h of coculture, and similar colocalization pattern of HRP and ITGB1 was observed at 12 h, 18 h, and 24 h of coculture (supplemental Fig. S4). CDCP1 and ITGA6, which were identified in the cell–cell interactomes of only MDA-MB-231 cells, were also examined by imaging coculture of MCF7 HRP-TM and MDA-MB-231 cells. As expected, their localizations were found to be specific to MDA-MB-231 cells (Fig. 5, *B* and *C*), which suggests that both CDCP1 and ITGA6 are involved in the heterotypic interaction of MDA-MB-231 with MCF7. Collectively, our findings from the proteomic study (Fig. 4*B*) were confirmed by imaging cocultures (Fig. 5).

### Five Signaling Pathways are Predominant in Homotypic and Heterotypic Interactions

**Fig. 6:**
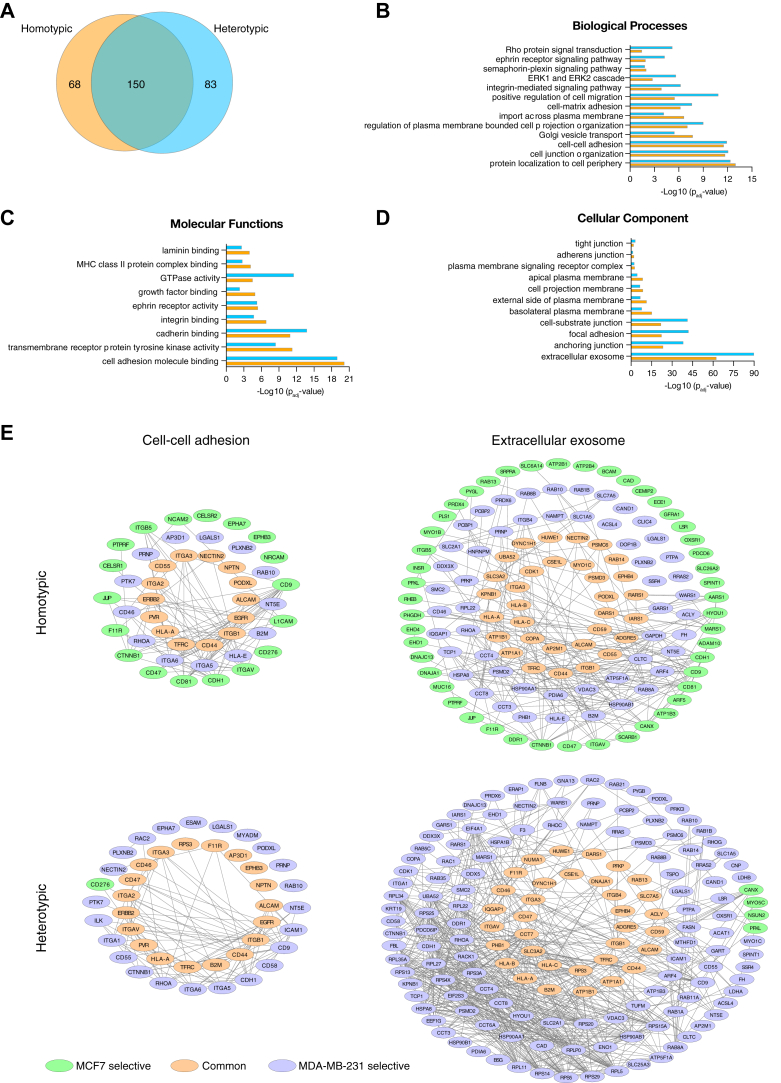
**The GO enrichment analysis of epithelial and mesenchymal proteome at homotypic and heterotypic cell interactions.***A*, Venn diagram shows the number of common, homotypic, and heterotypic selective proteins from both epithelial and mesenchymal cells. The comparison of representative enriched GO terms for biological processes (*B*), molecular functions (*C*), and cellular component (*D*) between homotypic and heterotypic interactions. All enriched GO terms are listed in supplemental Table S3. The *p*-values of GO terms were corrected with Bonferroni step down by ClueGo. *E*, the subinteraction networks of cell-cell adhesion (GO:0098609) and extracellular exosome (GO:0070062) as representative for biological processes and cellular component, respectively. The rest of the networks are presented in supplemental Figs. S5–S7. The protein–protein interactions were retrieved from the STRING database to generate subinteraction networks using Cytoscape.

When we compared the homotypic and heterotypic interactome regardless of origin of cell, including both epithelial and mesenchymal proteomes, we observed more than 60% overlapping proteins (Fig. 6*A*). To characterize the proteome of homotypic and heterotypic cell interactions, we performed GO enrichment analysis of epithelial and mesenchymal proteomes together, based on the biological processes, molecular functions, and CCs (Fig. 6, *B*–*D* and supplemental Table S3). The generation of subnetworks allowed us to reconstruct the interactions of common, MCF7 and MDA-MB-231 selective proteins in representative enriched GO terms and to map the differences between homotypic and heterotypic interactions (Fig. 6*E* and supplemental Figs. S5–S7).

We focused on the biological processes such as cell–cell and cell–matrix adhesion, cell junction organization, and the regulation of cell migration because of their importance in collective invasion in cancer progression. Interestingly, the heterotypic interactome was more enriched in the positive regulation of cell migration in comparison to homotypic interactome, which supports the role of heterotypic cell interactions in collective migration. Higher number of transport proteins such as solute carrier proteins and ATPases that coordinates the entrance and exit of metabolites and ions across the plasma membrane (47) were identified in homotypic interactome. This might be due to the tight interaction and communication of same cells. Similarly, homotypic interactome was more enriched in the Golgi-vesicle transport, which involves the trafficking of proteins from the Golgi to the plasma membrane and extracellular space (48). As signaling pathways are important biological processes to transform the extracellular signals into a wide range of intracellular responses, we also aimed to manifest the changes in these networks. Proteins at homotypic and heterotypic interactions were enriched in five signaling pathways, more strongly in the latter. 1: Integrin-mediated signaling pathway that is linked to various stages of cancer progression (49), 2: ERK1 and ERK2 cascade that drives major cellular processes including cell motility upon extracellular signals (50), 3: Semaphorin–plexin signaling pathway that remodels cytoskeleton and regulates cell adhesion (51), 4: Ephrin receptor signaling cascade that allows short distance cell–cell communication to regulate the cell motility and morphology (52), and 5: Rho protein signal transduction that is involved in the regulation of cell shape changes, cytokinesis, cell adhesion, and cell migration (53) (Fig. 6*B* and supplemental Fig. S5). Additionally, we noticed that the transmembrane receptor protein tyrosine kinase signaling pathway, which is essential for various cellular process such as cell proliferation, migration, and differentiation (54) was only significant in the homotypic interactome (supplemental Table S3).

We also compared the homotypic and heterotypic interactomes based on the molecular functions such as cell adhesion molecule binding, transmembrane receptor protein tyrosine kinase activity, cadherin, integrin and growth factor binding, and GTPase activity that are important for cell–cell and cell–matrix adhesion (Fig. 6*C* and supplemental Fig. S6). The most prominent difference between homotypic and heterotypic interactomes was observed for GTPase activity, which coordinates multiple signaling pathways using a molecular switch mechanism (53). The greater extent of enrichment in both GTPase activity and signaling pathways for heterotypic interactions might suggest the activation of signaling transduction upon coculture of different cell types. Intriguingly, extracellular exosomes were the most enriched cellular component term for both homotypic and heterotypic interactions (Fig. 6, *D* and *E*). Extracellular exosomes appear as another mechanism of intercellular communication beside the classical contact-dependent cell–cell interaction. They may transfer bioactive molecules including membrane receptors, lipids, proteins, and even genetic information such as mRNA and miRNA to the target cells (55, 56). Besides, so many ribosomal proteins were identified in extracellular exosomes of MDA-MB-231 in heterotypic interactions. In line with this, 44 proteins were clustered for translation as a biological process selectively for only heterotypic interactions (supplemental Table S3). Following the extracellular exosomes, anchoring junction, focal adhesion, and cell-substrate junction proteins were more prominent in both homotypic and heterotypic interactions (Fig. 6*D* and supplemental Fig. S7).

### Proximity Labeling in 3D Coculture

**Fig. 7:**
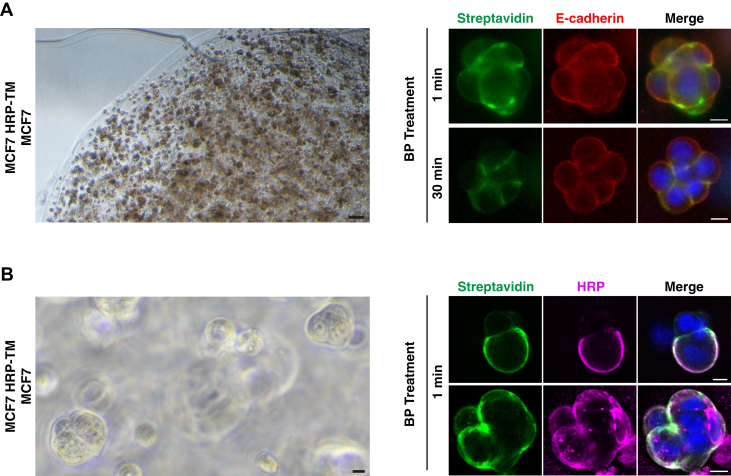
**Proximity labeling in 3D coculture.** Representative images of spheroids that were generated by encapsulating MCF7 HRP-TM and WT MCF7 cells using calcium alginate. *A*, the bright-field image reveals the edge of an alginate hydrogel bead and encapsulated spheroids at low magnification. Scale bar represents 100 μm (*left*). Spheroids were either preincubated with BP for 30 min or not. After proximity labeling for 1 min, spheroids were fixed and stained with fluorescent streptavidin for biotinylation and with anti-E-cadherin antibody to visualize MCF7 cells. DNA was stained with DAPI. Scale bars represent 10 μm (*right*). *B*, the bright-field image shows the encapsulated spheroids at high magnification. Scale bar represents 10 μm (*left*). After biotinylation for 1 min, spheroids were fixed and stained with fluorescent streptavidin for biotinylation and with anti-HRP antibody to visualize MCF7 HRP-TM cells. DNA was stained with Hoescht. Two representative fluorescent images of spheroids are shown. Scale bars represent 10 μm (*right*).

Based on our results above, we concluded that we established a robust assay to decode the protein composition of cell–cell interactions in 2D coculture. Next, we asked whether this method can be applied to 3D coculture. For this, we tested HRP-dependent biotinylation of the proteins at cell–cell interactions in 3D environment. Alginate, as an inert biopolymer, offers a biocompatible and tunable scaffold for culturing cells within a 3D environment. Its broad adoption as a natural polymer facilitates diverse experimental methodologies, including the exploration of cellular interactions, growth dynamics, proliferation, and differentiation processes (57, 58). We encapsulated both HRP-TM–expressing and WT MCF7 cells in alginate hydrogel beads *via* CaCl_2_-based ionic crosslinking and cultured for 18 h to form spheroids (Fig. 7*A*). To ensure the delivery of BP inside the spheroid, we pretreated spheroids with BP for 30 min before starting the labeling reaction by adding H_2_O_2_. In parallel, spheroids without any long BP preincubation were set as control. Immunofluorescence analysis revealed that HRP-dependent biotinylation with no prior BP incubation was sufficient to label the interaction sites (Fig. 7*A*). We also showed that the biotinylation was specific to HRP-TM–expressing cells and was not detectable in WT cells (Fig. 7*B*). This result suggests that the methodology is not restricted to only 2D coculture but can also be applied to 3D models.

## Discussion

In this study, we developed a novel method which combines the enzyme-catalyzed proximity labeling in live cells and a SILAC-based proteomic approach to identify the proteins at the interface of homotypic and heterotypic cancer cell interactions.

This methodology exploited contact-independent labeling, which allowed to identify not only the membrane-associated cell surface proteins of neighboring cells but also their secreted soluble factors and exosomal proteins in the extracellular environment. As a proof of principle for the methodology, we chose the HRP-conjugated transmembrane domain of platelet-derived growth factor receptor (HRP-TM) (16), which successfully covers the whole cell surface as labeling agent. In general, any protein of interest on the cell surface can be fused with HRP to identify its interaction partners from neighboring cells.

Although we implemented this methodology to examine epithelial–mesenchymal cancer cell interactions, the application of this integrative proteomic approach can be extended to study any type of cell–cell interaction such as cancer–stromal cell or neuron–glia interaction. However, it should be noted that the cell size, morphology, and adhesion properties of bait and prey cells in heterotypic cocultures may affect the labeling efficiency of cell–cell interactions. In our study, we observed higher biotinylation, thus identified more proteins in heterotypic interaction of MDA-MB-231 compared to MCF7 cells. This is due to the differences in the nature of bait cells and so, HRP levels between heterotypic cocultures of MCF7 and MDA-MB-231 cells. Although our proteomic study was performed to profile cell–cell interactome in 2D coculture system, we also demonstrated that this method can be extended to 3D coculture models. By imaging, we showed that cocultured spheroids can be formed in 18 h and the cell interaction sites of spheroids can be efficiently proximity labeled. In addition, mass spectrometry–based proteomic analysis of multicellular tumor spheroids is well-established (59, 60). Therefore, the proteomic analysis following the proximity labeling would be the next step to investigate cell–cell interactions in 3D models.

Our analysis unveiled the protein composition of the homotypic and heterotypic interactions of epithelial and mesenchymal breast cancer cells: a diverse set of proteins with various functional categories such as cell surface receptors, adhesion molecules, and components of signaling cascades. It revealed the prominent involvement of five signaling pathways across both homotypic and heterotypic interactions: the integrin-mediated signaling pathway, the ERK1 and ERK2 cascade, Semaphorin–plexin signaling pathway, Ephrin receptor signaling pathway, and Rho protein signal transduction. These pathways are integral to cellular processes governing adhesion, proliferation, and motility, implicating their significance in the context of cell–cell interactions within the tumor microenvironment. A notable enrichment of extracellular exosomes underscores their vital role in intercellular communication within tumors. Our study provides a comprehensive catalog of proteins orchestrating cell–cell interactions. Elucidating the protein constituents and signaling pathways offers valuable insights into the underlying mechanisms.

Overall, this study presents a new pipeline of experimental protocols and strategies, along with bioinformatic analyses, that can be used to comprehensively examine the proteome of any type of cell–cell interaction. In this example, our large-scale biochemical analysis provides a potential resource for the molecular dissection of cancer cell interactions in the future.

## Data availability

The mass spectrometry data (.raw), database search files (.msf and .pdResult), and peak files (.mzML) have been deposited to the ProteomeXchange Consortium (http://proteomecentral.proteomexchange.org) *via* the PRIDE partner repository (61) with the dataset identifier PXD060134. The annotated spectra of single unique peptides can be viewed from the .pdResult files.

## Supplemental data

This article contains supplemental data.

## Conflict of interest

The authors declare no competing interests.
