# Supplementary figures and images for: Proximity Labeling and SILAC-Based Proteomic Approach Identifies Proteins at the Interface of Homotypic and Heterotypic Cancer Cell Interactions
Source: Mol Cell Proteomics. 2025 May 5;24(6):100986. doi: 10.1016/j.mcpro.2025.100986 (PMC12289527; doi:10.1016/j.mcpro.2025.100986)

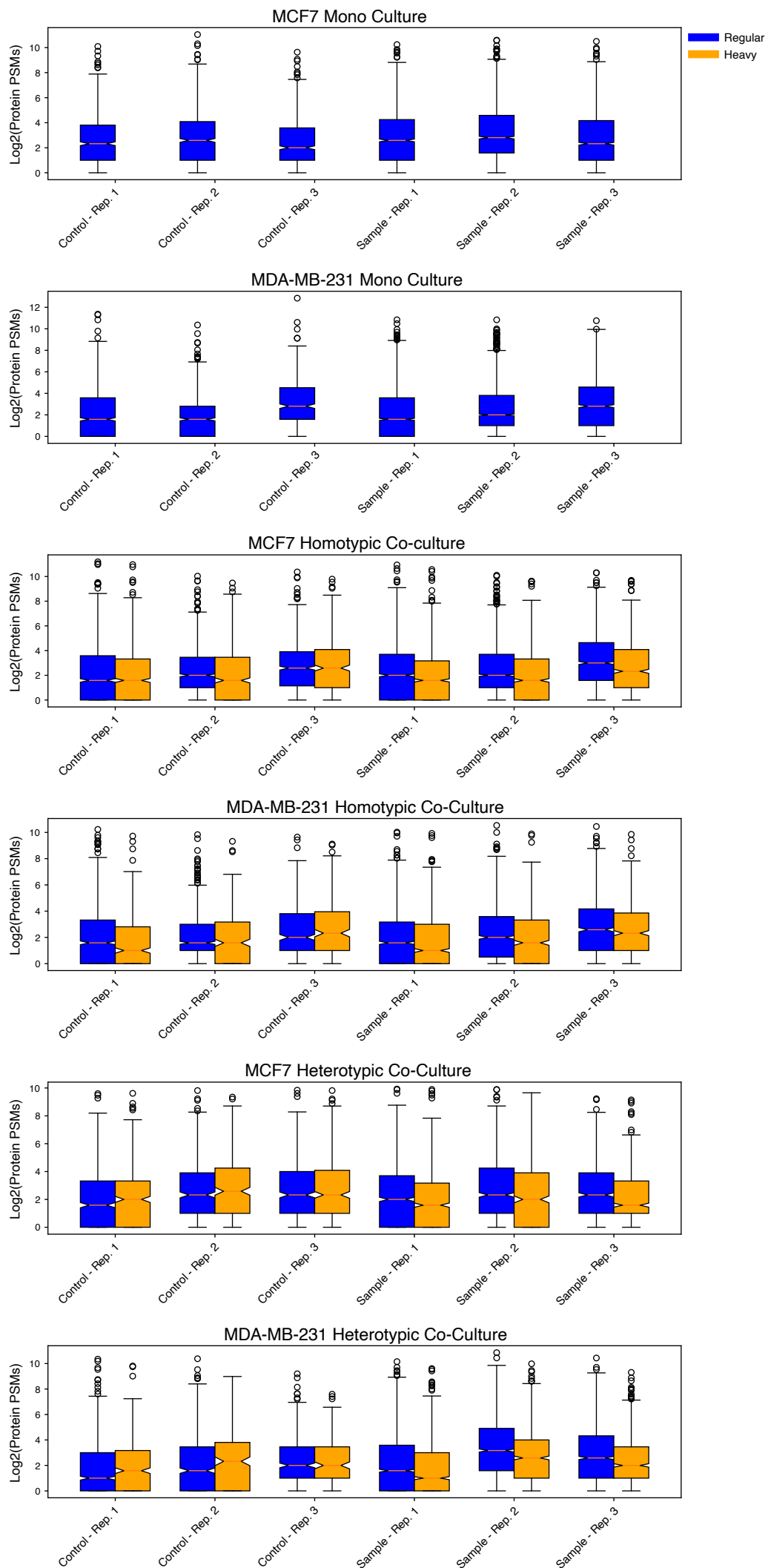

**Supplemental Fig. S1**

Supplement: Supplemental Fig.S1 [file mmc1.pdf]

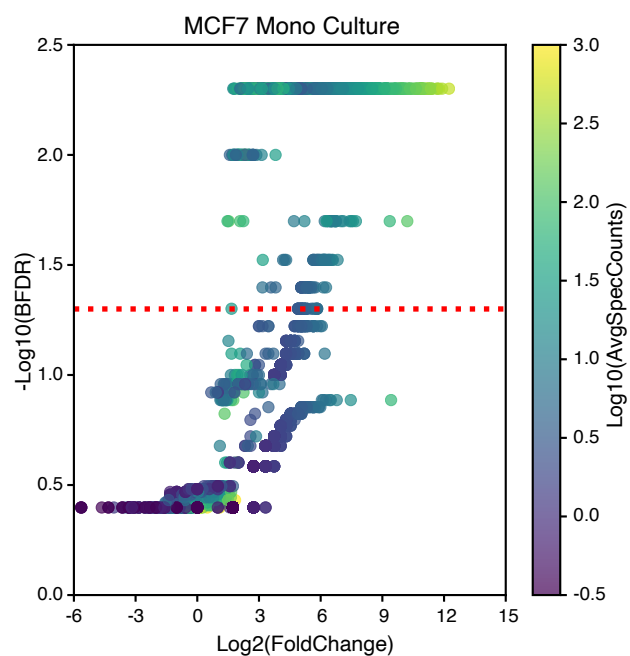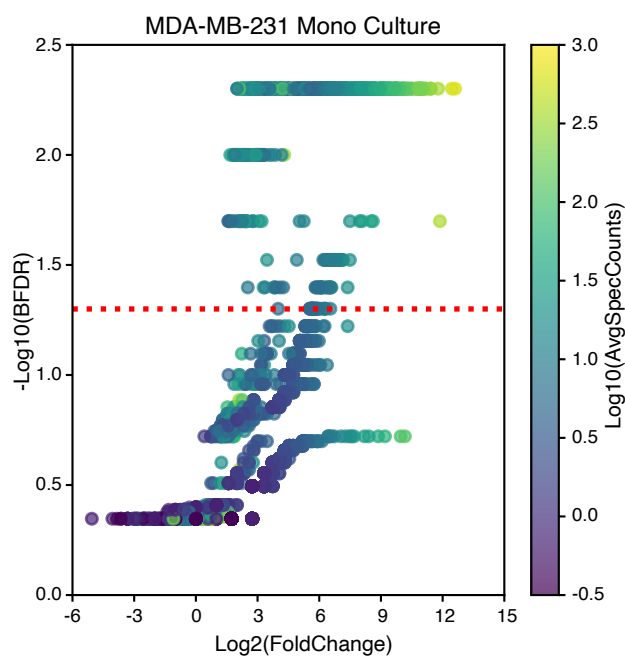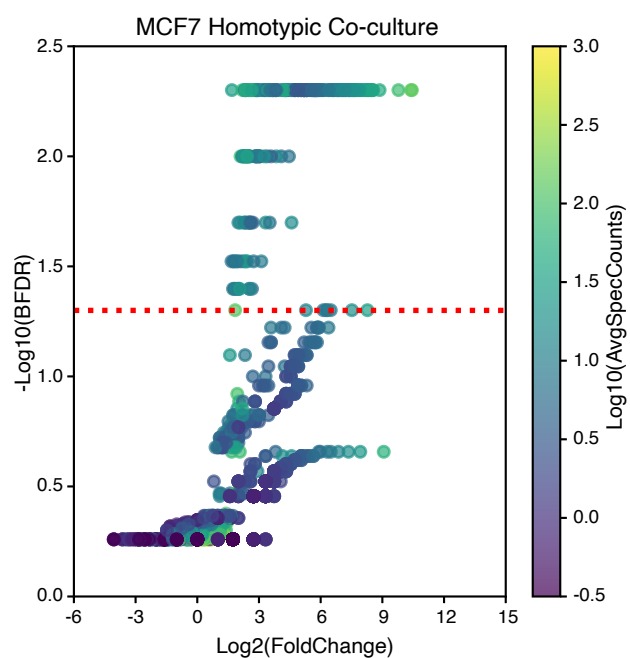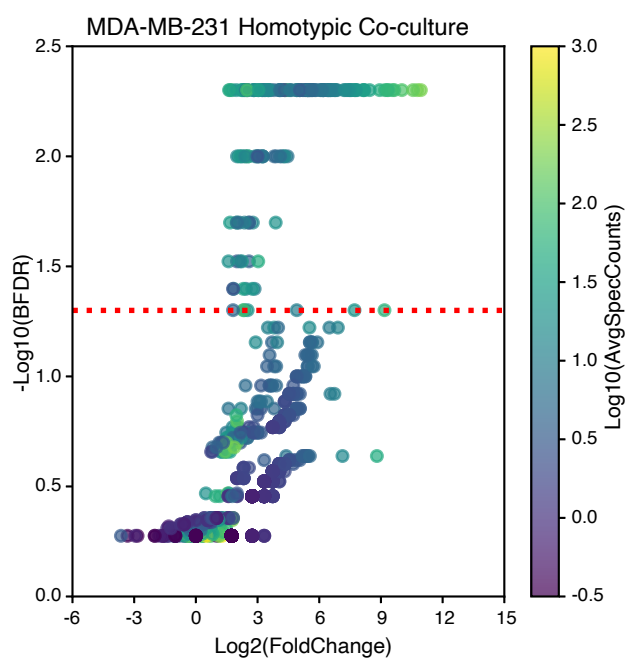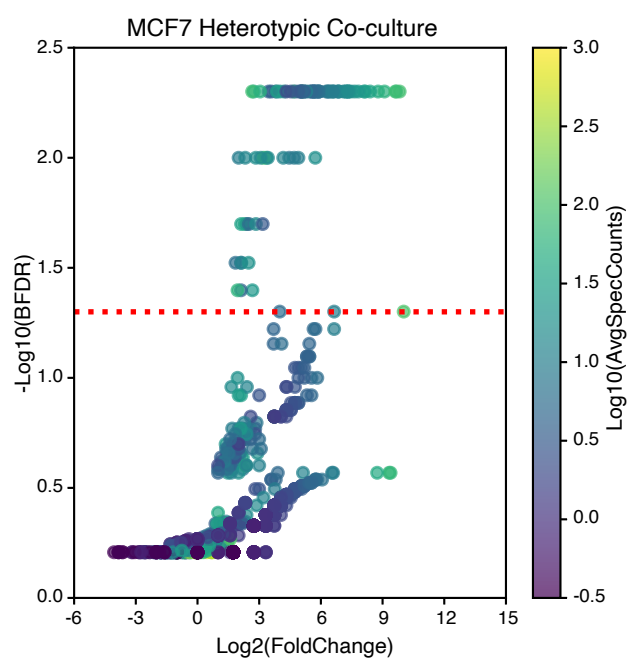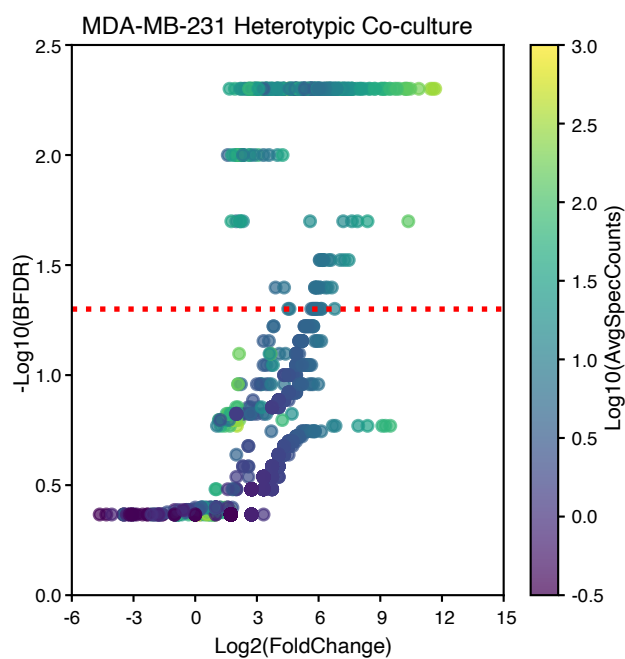

**Supplemental Fig. S2**

Supplement: Supplemental Fig.S2 [file mmc2.pdf]

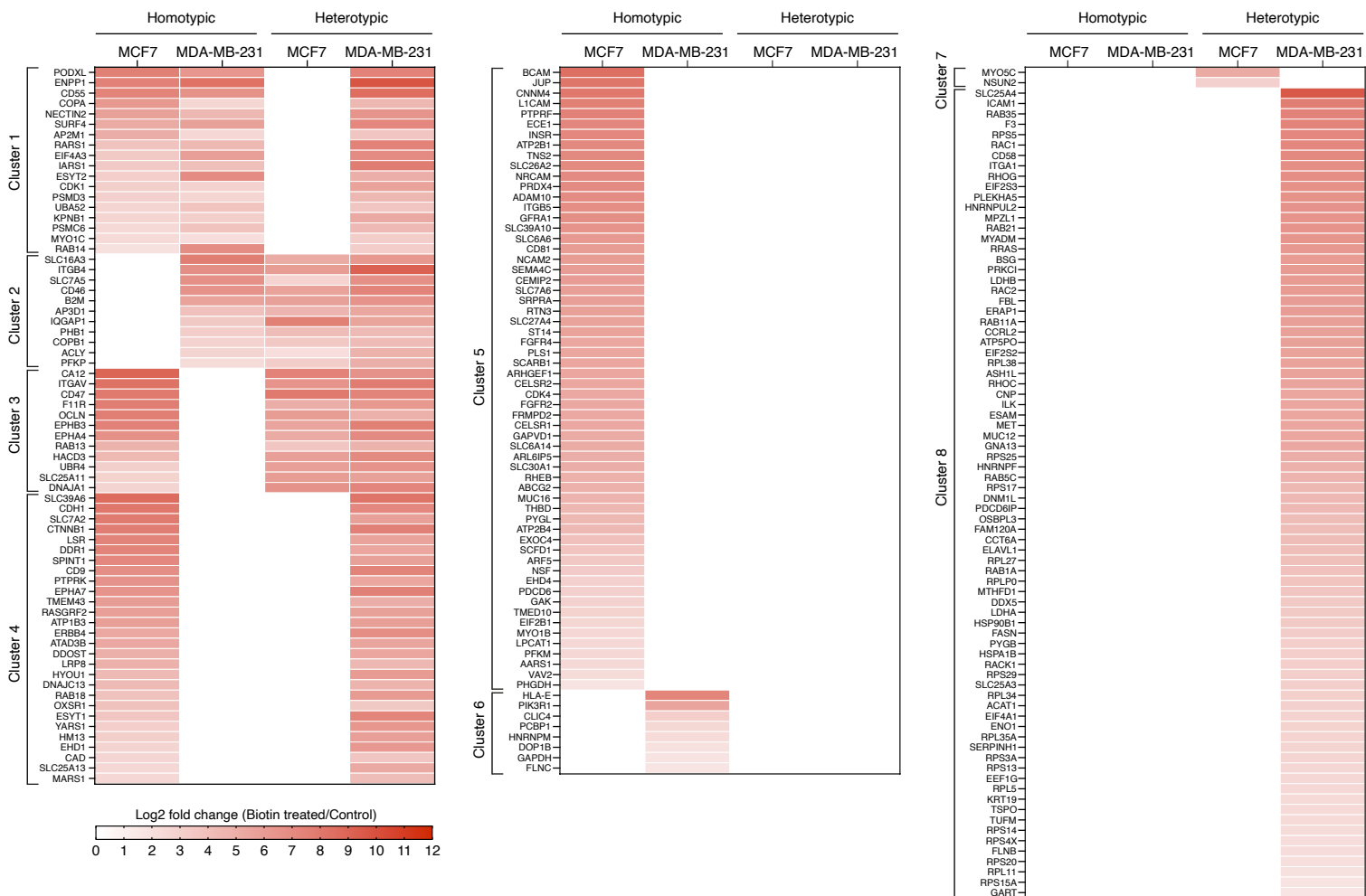

**Supplemental Fig. S3**

Supplement: Supplemental Fig.S3 [file mmc3.pdf]

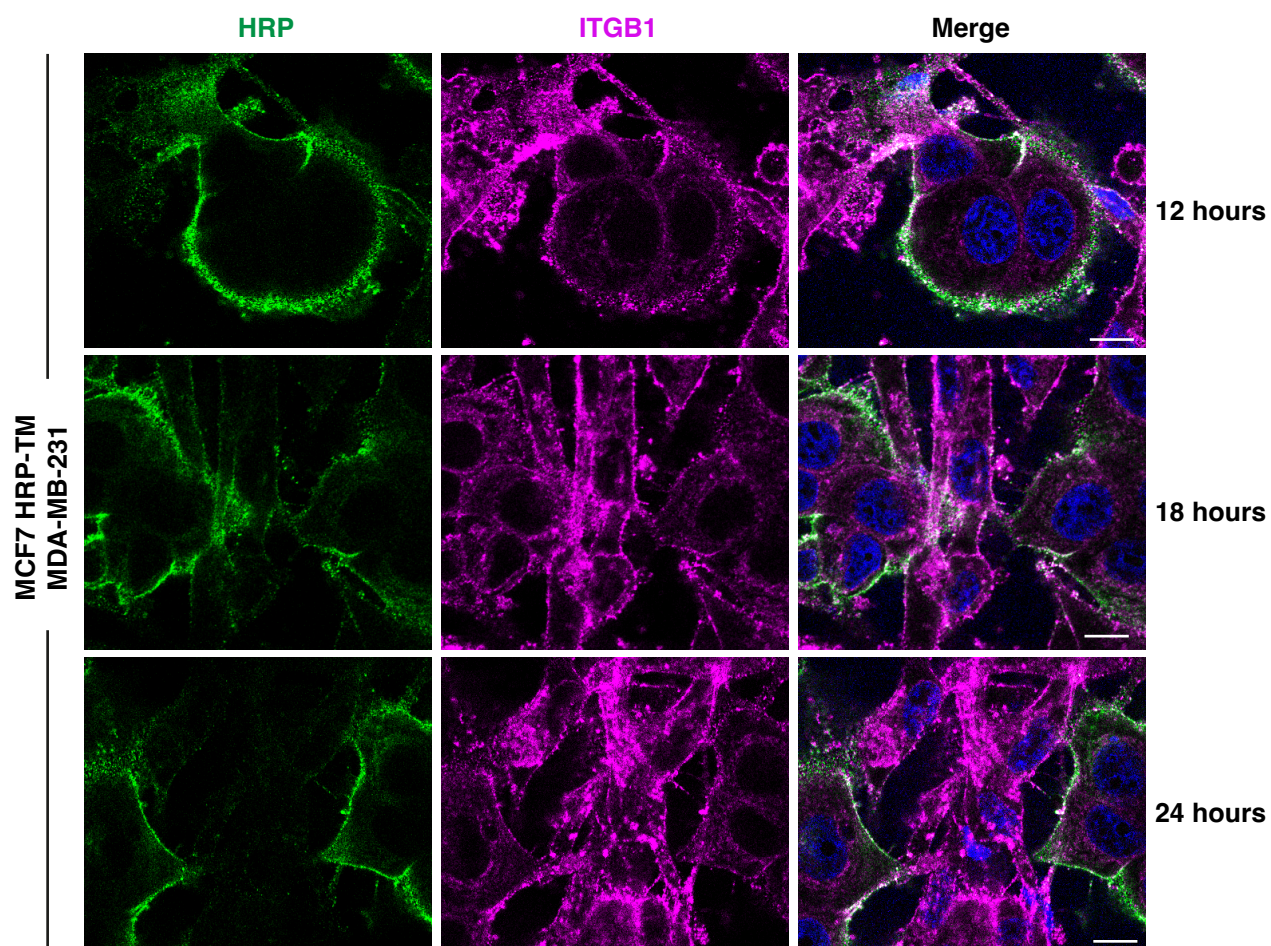

**Supplemental Fig. S4**

Supplement: Supplemental Fig.S4 [file mmc4.pdf]

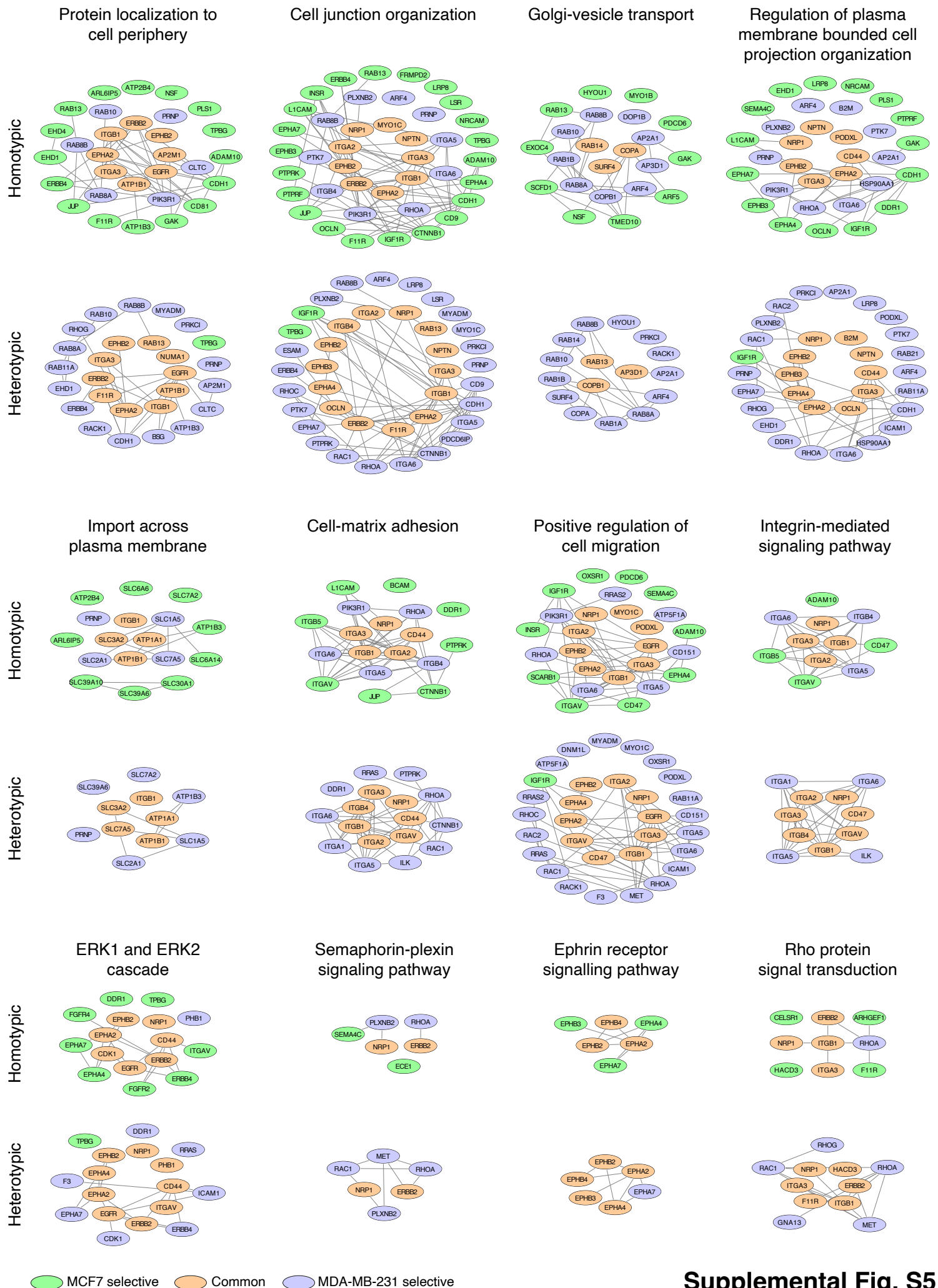

**Supplemental Fig. S5**

Supplement: Supplemental Fig.S5 [file mmc5.pdf]

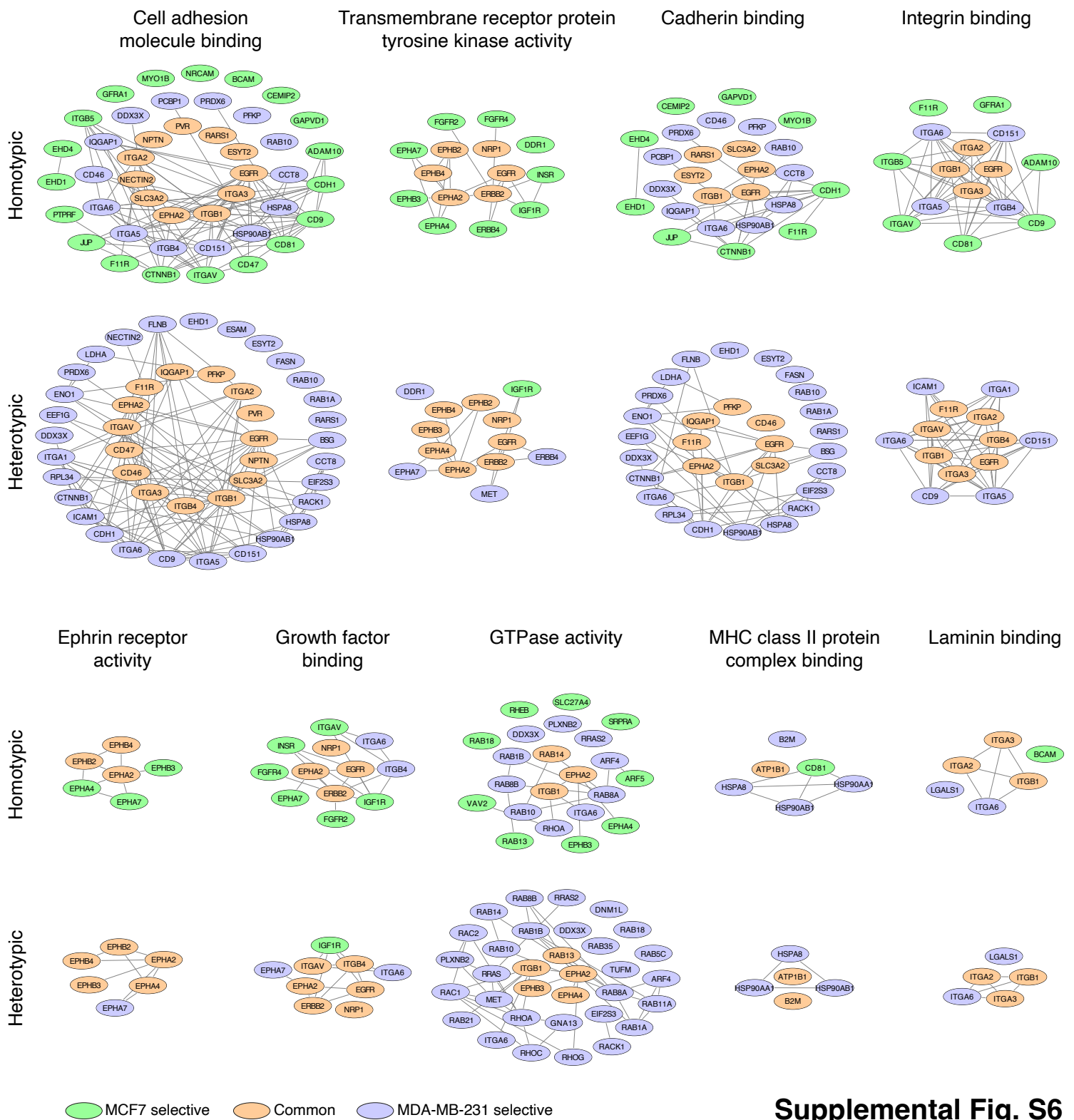

**Supplemental Fig. S6**

Supplement: Supplemental Fig.S6 [file mmc6.pdf]
