# Supplementary material for: Proximity Labeling and SILAC-Based Proteomic Approach Identifies Proteins at the Interface of Homotypic and Heterotypic Cancer Cell Interactions
Source: Mol Cell Proteomics. 2025 May 5;24(6):100986. doi: 10.1016/j.mcpro.2025.100986 (PMC12289527; doi:10.1016/j.mcpro.2025.100986)
Supplement: Supplemental Fig.S7 [file mmc7.pdf]

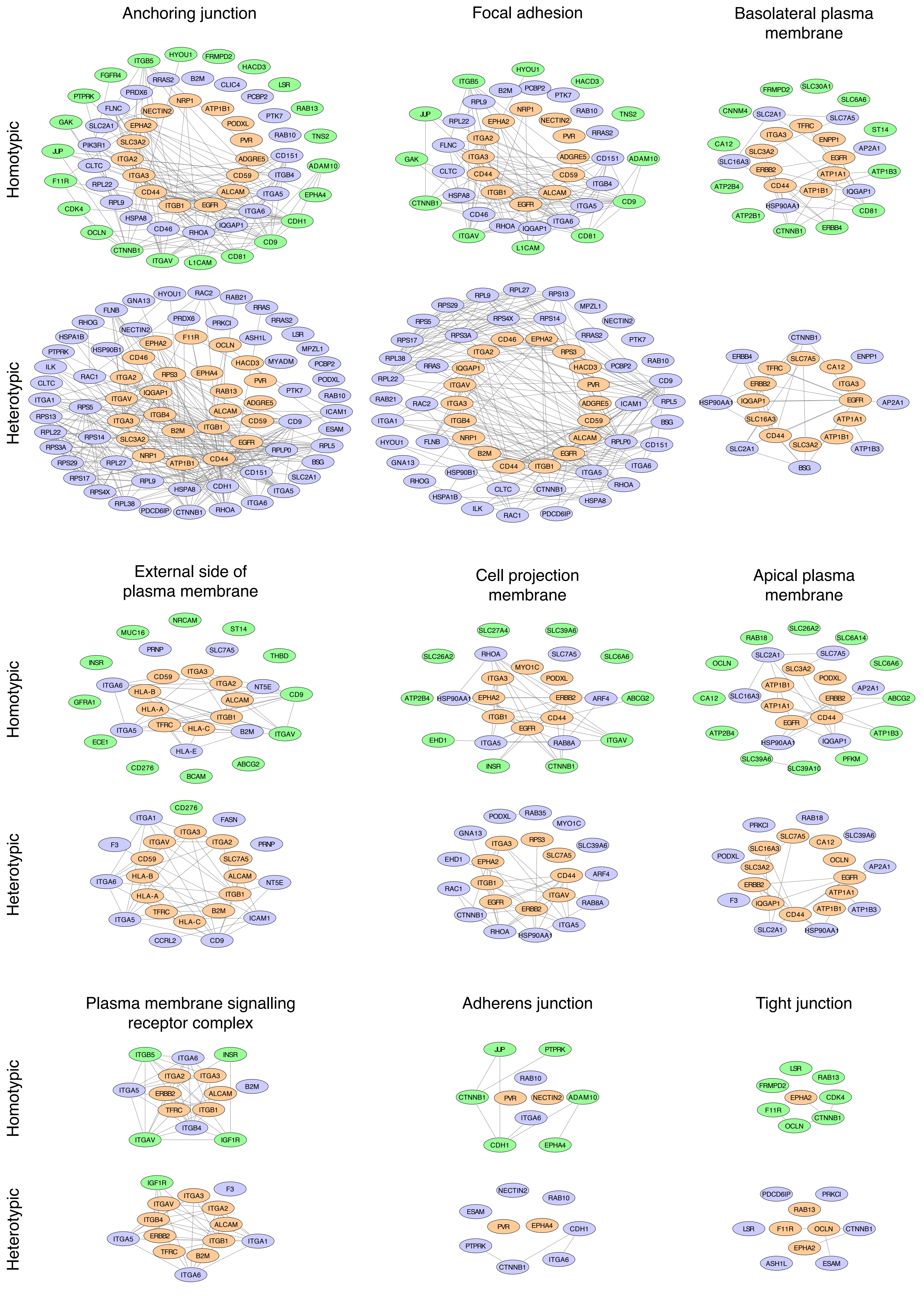

Supplemental Fig. S7
