# Supplementary material for: Proximity Labeling and SILAC-Based Proteomic Approach Identifies Proteins at the Interface of Homotypic and Heterotypic Cancer Cell Interactions
Source: Mol Cell Proteomics. 2025 May 5;24(6):100986. doi: 10.1016/j.mcpro.2025.100986 (PMC12289527; doi:10.1016/j.mcpro.2025.100986)
Supplement: Supplemental Data [file mmc11.pdf]

Proximity labeling and SILAC based proteomic approach identifies proteins at the interface of homotypic and heterotypic cancer cell interactions.

Nazan Saner[1*], Ceren Uzun[2#], Büşra Aytül Kırım[1], Sena Nur Özkan[3], Daniel Jon Geiszler[1], Ece Öztürk[3,4], Nurcan Tunçbağ[2], Nurhan Özlü[1,3*].

Supplemental Fig. S1. The overall distribution of spectral counts per protein for mono-cultures, homotypic and heterotypic co-cultures. PSM counts of peptides were summed up for each protein. Samples were treated with BP and H_2O_2 whereas controls with only H_2O_2.

Supplemental Fig. S2. The overview of SAINTexpress results showing the log fold-changes, mean spectral counts, and significance of all proteins for mono-cultures, homotypic and heterotypic co-cultures. Only heavy labelled proteins were included in SAINTexpress analysis of co-cultures. BFDR = 0.05 is represented by the dashed red line.

Supplemental Fig. S3. The clustering of proteins based on their identification in different breast cancer cell line and co-culture conditions. Supplemental to Fig. 4B. Heatmap reveals the log_2 fold changes of identified proteins in comparison to controls.

Supplemental Fig. S4. Time series of 12 h, 18 h and 24 h for the co-culture of MCF7 HRP and MDA-MB-231 cells reveal the establishment of cell-cell contacts. The representative immunofluorescence images of MCF7 HRP-TM and MDA-MB-231 cells that were co-cultured for 12 h, 18 h and 24 h. Cells were fixed and stained with anti-HRP and anti-ITGB1 antibodies. DNA was stained with Hoescht. Scale bar: 10 µm.

Supplemental Fig. S5. The sub-interaction networks of homotypic and heterotypic cancer cell interactions for enriched GO biological processes. The sub-interaction networks of protein localization to cell periphery (GO:1990778), cell junction organization

(GO:0034330), Golgi vesicle transport (GO:0048193), regulation of plasma membrane bounded cell projection organization (GO:0120035), import across plasma membrane (GO:0098739), cell-matrix adhesion (GO:0007160), positive regulation of cell migration (GO:0030335), integrin-mediated signaling pathway (GO:0007229), ERK1 and ERK2 cascade (GO:0070371), semaphorin-plexin signaling pathway (GO:0071526), ephrin receptor signaling pathway (GO:0048013) and Rho protein signal transduction (GO:0007266). Supplemental to Fig. 6E. The protein-protein interactions were retrieved from the STRING database to generate sub-interaction networks using Cytoscape.

Supplemental Fig. S6. The sub-interaction networks of homotypic and heterotypic cancer cell interactions for enriched GO molecular functions. The sub-interaction networks of cell adhesion molecule binding (GO:0050839), transmembrane receptor protein tyrosine kinase activity (GO:0004714), cadherin binding (GO:0045296), integrin binding (GO:0005178), ephrin receptor activity (GO:0005003), growth factor binding (GO:0019838), GTPase activity (GO:0003924), MHC class II protein complex binding (GO:0023026) and laminin binding (GO:0043236). Supplemental to Fig. 6E. The protein-protein interactions were retrieved from the STRING database to generate sub-interaction networks using Cytoscape.

Supplemental Fig. S7. The sub-interaction networks of homotypic and heterotypic cancer cell interactions for enriched GO cellular component. The sub-interaction networks of anchoring junction (GO:0070161), focal adhesion (GO:0005925), basolateral plasma membrane (GO:0016323), external side of plasma membrane (GO:0009897), cell projection membrane (GO:0031253), apical plasma membrane (GO:0016324), plasma membrane signaling receptor complex (GO:0098802), adherens junction (GO:0005912) and tight junction (GO:0070160). Supplemental to Fig. 6E. The protein-protein interactions were retrieved from the STRING database to generate sub-interaction networks using Cytoscape.

Supplemental Table S1. The list of all peptides identified in mono-cultures, homotypic and heterotypic co-cultures.

Supplemental Table S2. The list of high confidence proteins identified in mono-cultures, homotypic and heterotypic co-cultures.

Supplemental Table S3. The list of enriched GO terms for biological processes, molecular functions, and cellular component at homotypic and heterotypic cancer cell interactions.

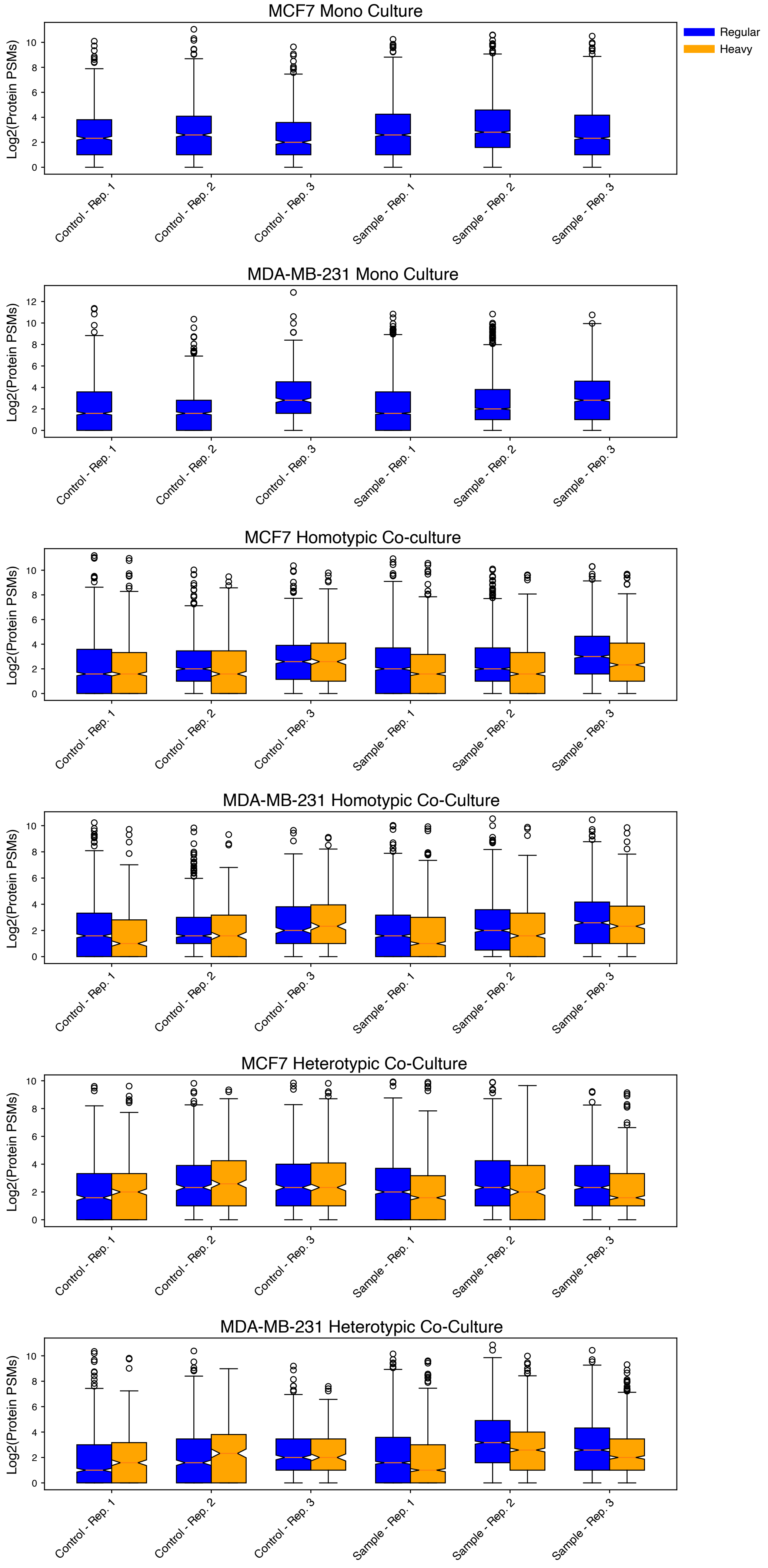

Supplemental Fig. S1

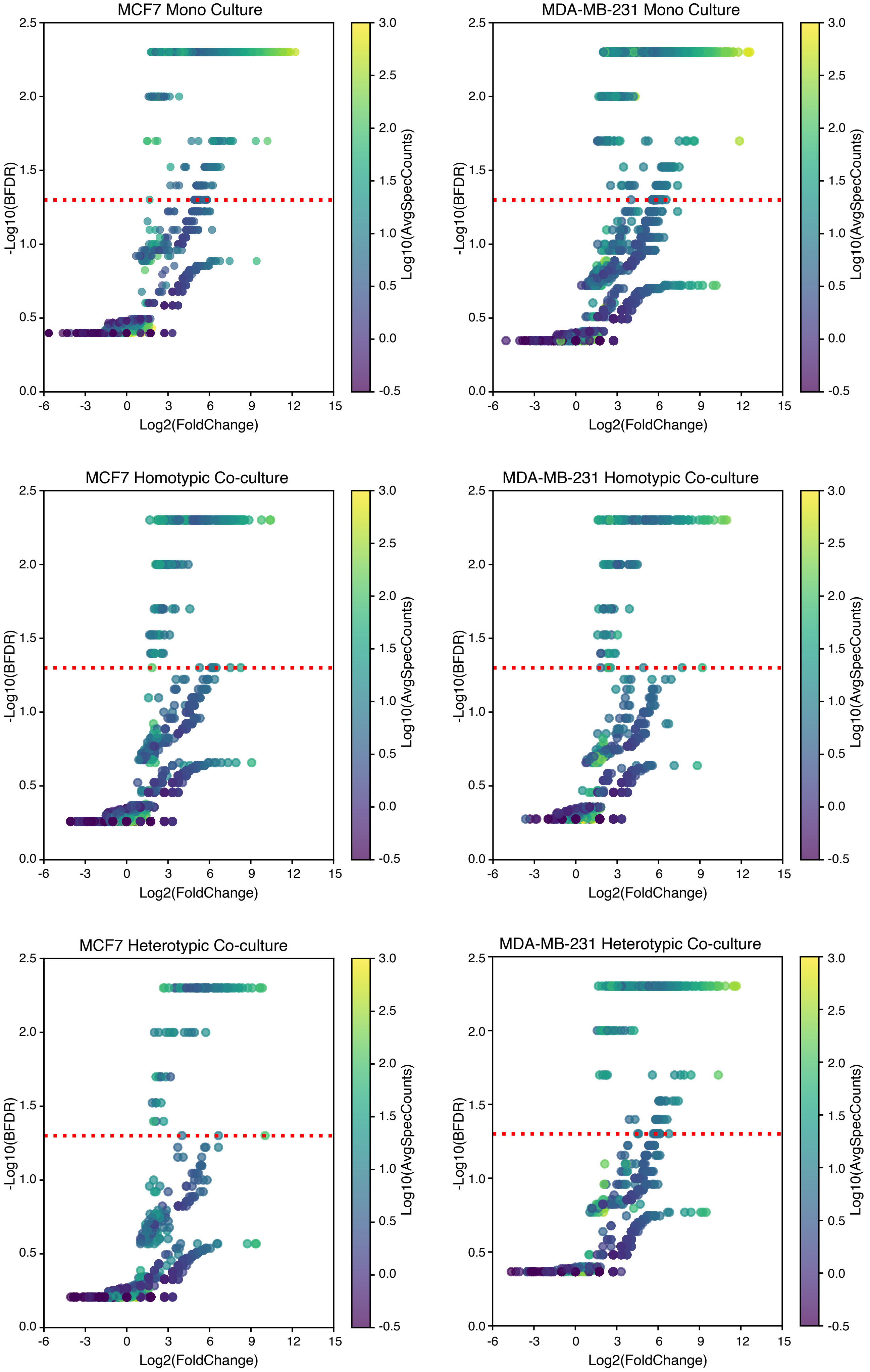

Supplemental Fig. S2

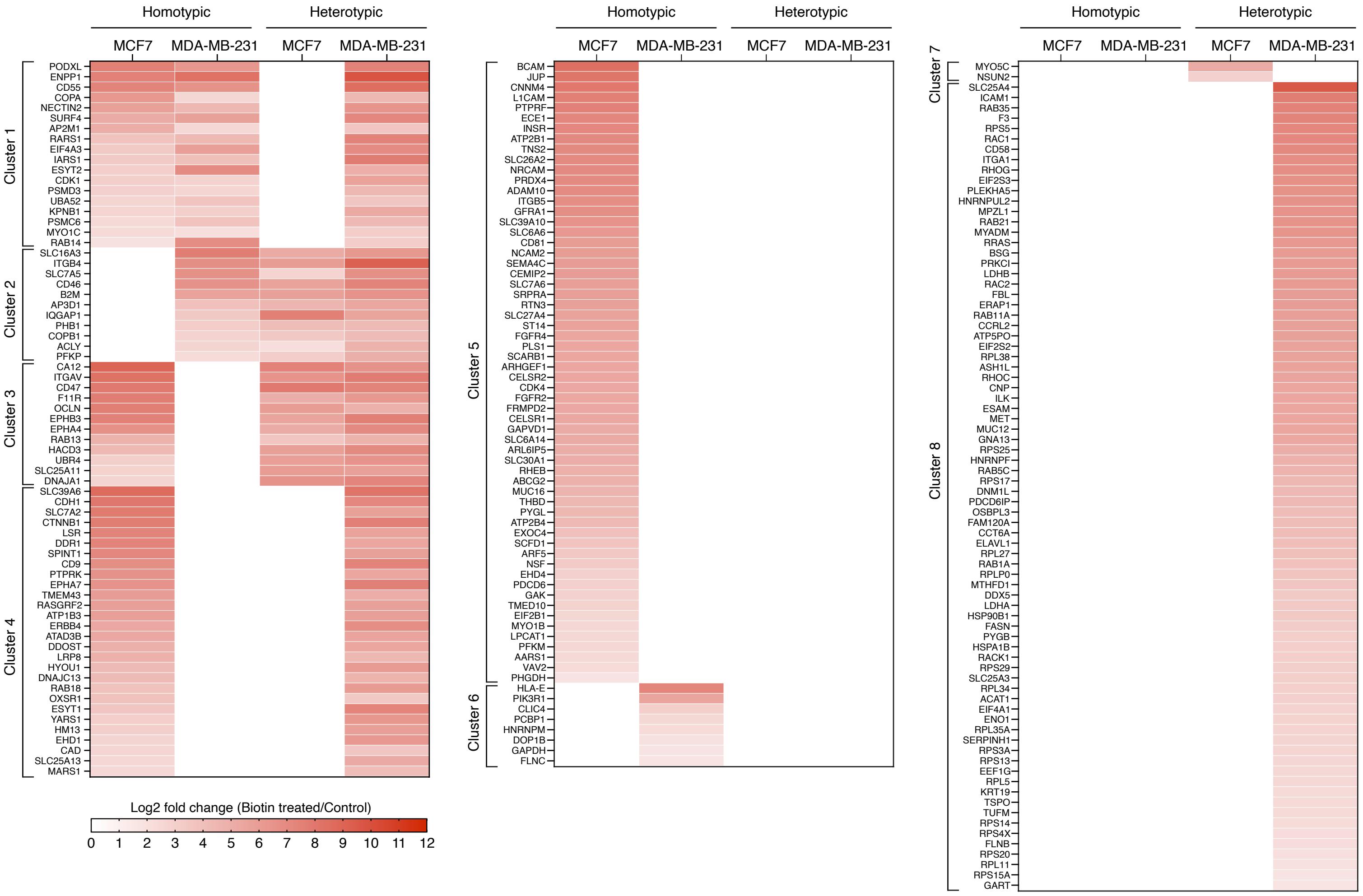

Supplemental Fig. S3

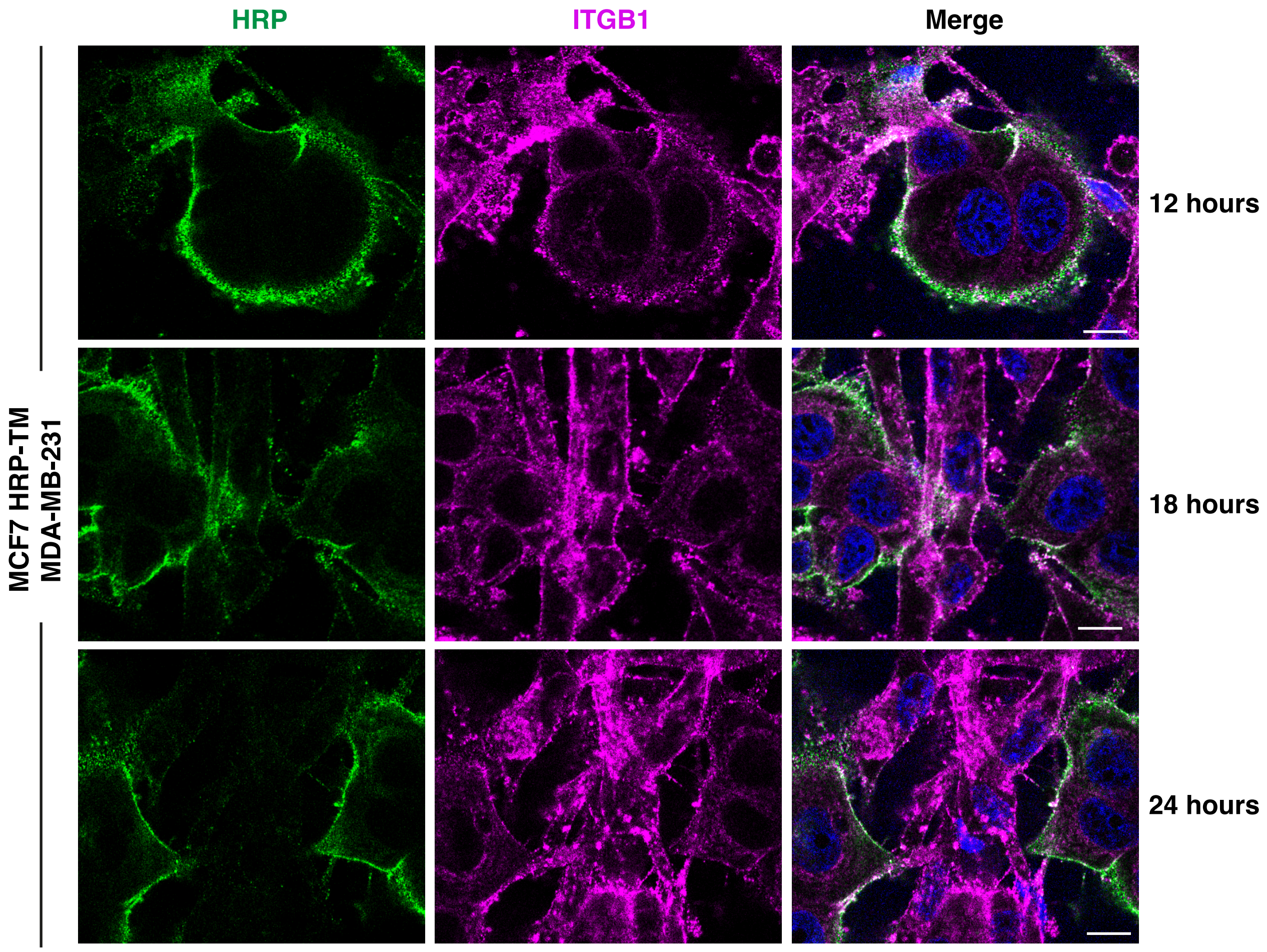

Supplemental Fig. S4

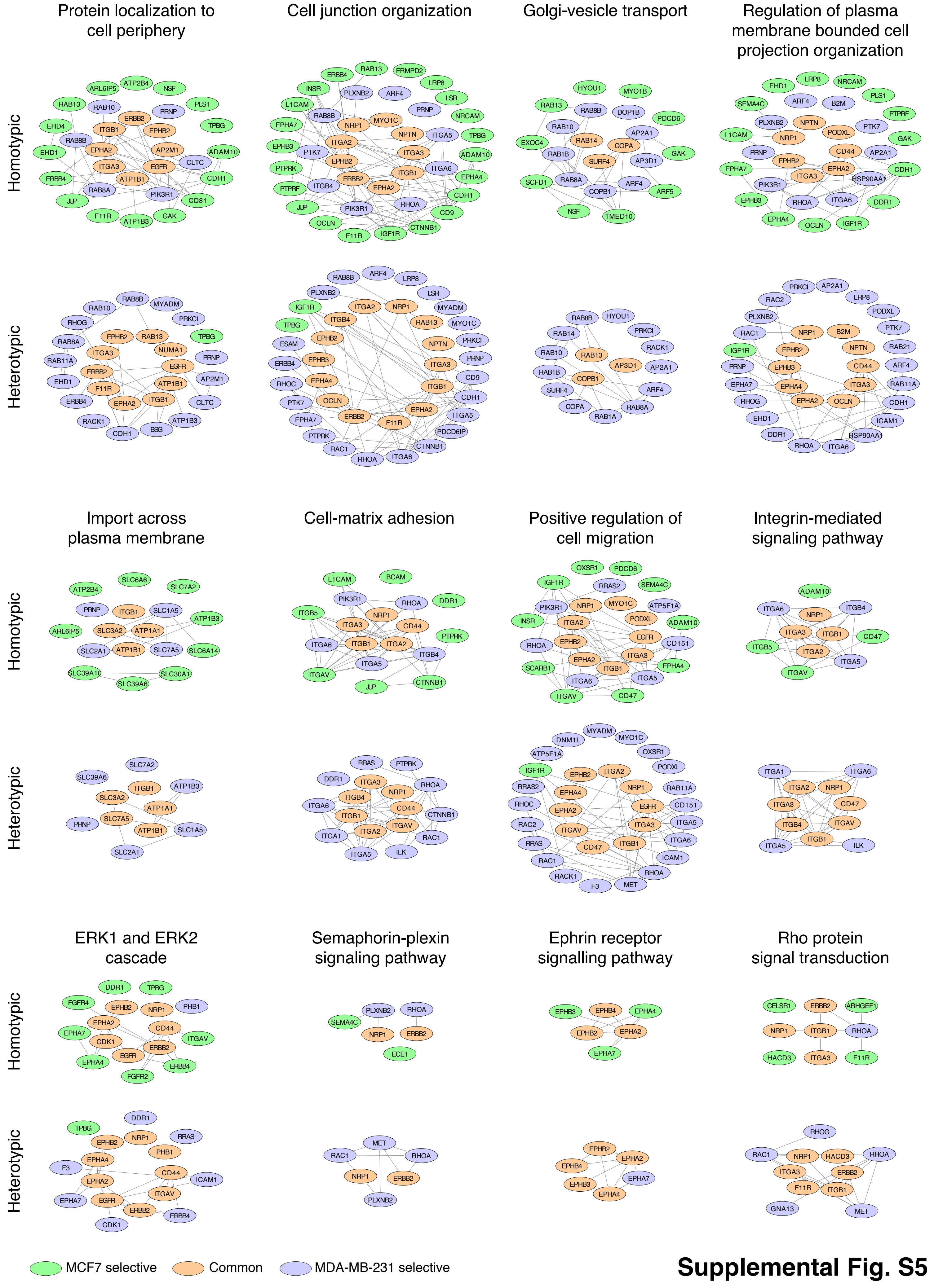

Supplemental Fig. S5

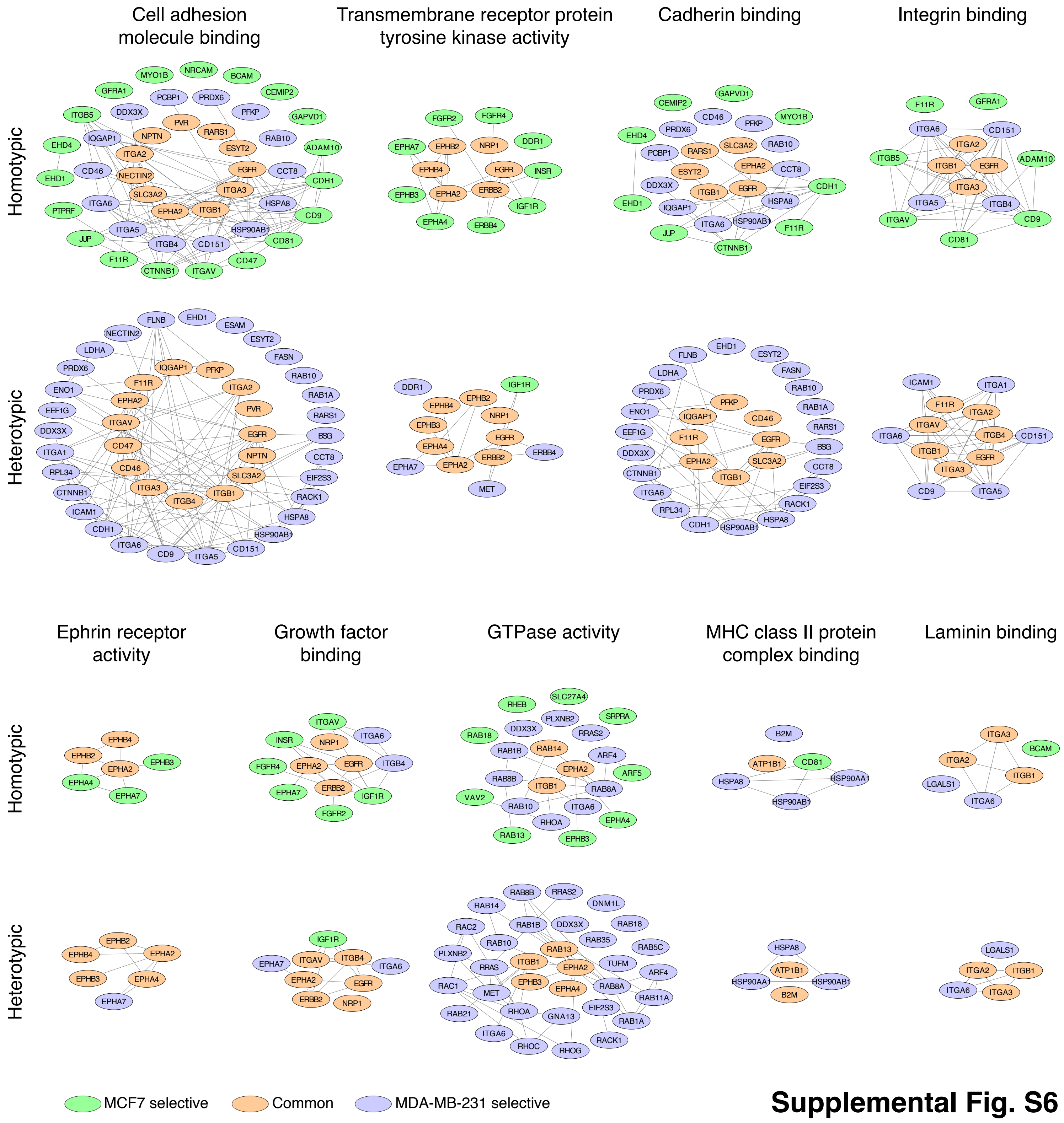

Supplemental Fig. S6

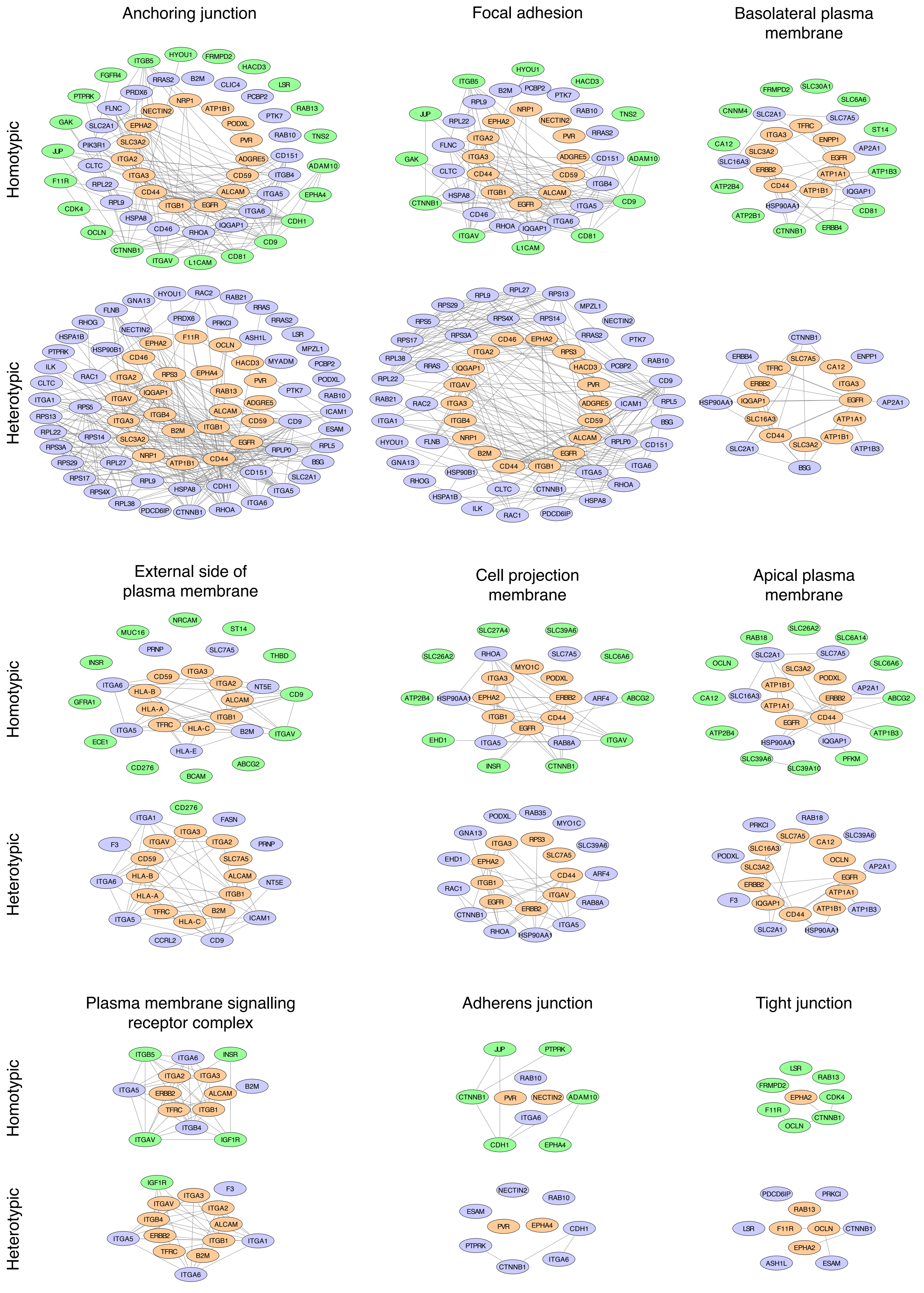

Supplemental Fig. S7
